# The role of microsporidian polar tube protein 4 (PTP4) in host cell infection

**DOI:** 10.1371/journal.ppat.1006341

**Published:** 2017-04-20

**Authors:** Bing Han, Valérie Polonais, Tatsuki Sugi, Rama Yakubu, Peter M. Takvorian, Ann Cali, Keith Maier, Mengxian Long, Matthew Levy, Herbert B. Tanowitz, Guoqing Pan, Frédéric Delbac, Zeyang Zhou, Louis M. Weiss

**Affiliations:** 1 State Key Laboratory of Silkworm Genome Biology, Southwest University, Chongqing, P. R. China; 2 Department of Pathology, Albert Einstein College of Medicine, Bronx, New York, United States of America; 3 Key Laboratory for Sericulture Functional Genomics and Biotechnology of Agricultural Ministry, Southwest University, Chongqing, P. R. China; 4 Université Clermont Auvergne, Laboratoire "Microorganismes: Génome et Environnement, Clermont-Ferrand, France; 5 CNRS, UMR 6023, LMGE, Aubière, France; 6 Department of Biological Sciences, Rutgers University, Newark, New Jersey, United States of America; 7 Department of Biochemistry, Albert Einstein College of Medicine, Bronx, New York, United States of America; 8 Department of Medicine, Albert Einstein College of Medicine, Bronx, New York, United States of America; 9 College of Life Sciences, Chongqing Normal University, Chongqing, P. R. China; University of California, San Diego, UNITED STATES

## Abstract

Microsporidia have been identified as pathogens that have important effects on our health, food security and economy. A key to the success of these obligate intracellular pathogens is their unique invasion organelle, the polar tube, which delivers the nucleus containing sporoplasm into host cells during invasion. Due to the size of the polar tube, the rapidity of polar tube discharge and sporoplasm passage, and the absence of genetic techniques for the manipulation of microsporidia, study of this organelle has been difficult and there is relatively little known regarding polar tube formation and the function of the proteins making up this structure. Herein, we have characterized polar tube protein 4 (PTP4) from the microsporidium *Encephalitozoon hellem* and found that a monoclonal antibody to PTP4 labels the tip of the polar tube suggesting that PTP4 might be involved in a direct interaction with host cell proteins during invasion. Further analyses employing indirect immunofluorescence (IFA), enzyme-linked immunosorbent (ELISA) and fluorescence-activated cell sorting (FACS) assays confirmed that PTP4 binds to mammalian cells. The addition of either recombinant PTP4 protein or anti-PTP4 antibody reduced microsporidian infection of its host cells *in vitro*. Proteomic analysis of PTP4 bound to host cell membranes purified by immunoprecipitation identified transferrin receptor 1 (TfR1) as a potential host cell interacting partner for PTP4. Additional experiments revealed that knocking out TfR1, adding TfR1 recombinant protein into cell culture, or adding anti-TfR1 antibody into cell culture significantly reduced microsporidian infection rates. These results indicate that PTP4 is an important protein competent of the polar tube involved in the mechanism of host cell infection utilized by these pathogens.

## Author summary

Microsporidia are obligate intracellular parasites that cause disease in immune suppressed individuals such as those with HIV/AIDS and recipients of organ transplants. The microsporidia are defined by a unique invasion organelle, the polar tube. The formation of this organelle and its role in the mechanism of infection remain unknown. Herein, we have identified a role for *Encephalitozoon hellem* polar tube protein 4 (PTP4) in infection demonstrating that PTP4 can bind to the host cell surface via the host transferrin receptor 1 (TfR1) protein. Interfering with the interaction of PTP4 and TfR1 causes a significant decrease in microsporidian infection of host cells. These data suggest that PTP4 functions as an important microsporidian protein during host cell infection by this pathogen.

## Introduction

Since the first microsporidium, *Nosema bombycis*, was discovered in the European silkworm industry in the 19th century [1], more than 1400 species of microsporidia have been identified worldwide [2, 3]. They are ubiquitous obligate intracellular parasites responsible for a variety of diseases both in immune compromised and immune competent individuals. Microsporidia are also responsible for economic losses due to their adverse effects on farming and other industries [4, 5]. Phylogenetic studies suggest that microsporidia are related to fungi, being either a basal branch or sister group [6–13].

Microsporidia have multiple transmission routes including oral transmission of spores through contaminated food and water [14, 15], and vertical transmission [16, 17]. They can infect a wide variety of animals ranging from invertebrate to vertebrate hosts, including humans and insects of economic importance such as the silkworm and honey bee [8, 18]. *Encephalitozoon hellem* is found in humans and was initially isolated from corneal biopsies and conjunctival scrapings from patients with advanced HIV-1 infection with keratoconjunctivitis [19]. Similar to other members of the family Encephalitozoonidae, *E*. *hellem* has been demonstrated to cause disseminated infection presenting with diarrhea, nephritis, keratitis and/or sinusitis [20–22]. Microsporidia possess a unique, highly specialized invasion mechanism that involves the polar tube and spore wall [23]. Despite the description of these pathogens 150 years ago [1], the mechanism of host cell invasion, the structure and formation of both the polar tube infection apparatus and invasion synapse, and the role of microsporidian-specific proteins during the invasion process are not understood.

The polar tube is a highly specialized invasion organelle. Before germination, the polar tube coils around the sporoplasm in the spore [24, 25]. Upon appropriate environmental stimulation, the polar tube will rapidly discharge out of the spore and then interact with and pierce a cell membrane serving as a conduit for the nucleus and sporoplasm passage into the host cell (the entire process taking place in <2 seconds) [26–28]. Since the initial description of the polar tube by Thelohan 100 years ago [24, 25], proteomic and antibody studies have led to the identification of five different polar tube proteins (PTP1 through PTP5) in microsporidia [29–33]. Analysis of protein glycosylation has revealed that PTP1 contains many post translational O-linked mannosylation sites and that these residues can bind concanavalin A (conA) [34, 35]. Pre-treatment of a host cell with mannose has been demonstrated to reduce the infectivity of *E*. *hellem*, which is consistent with an interaction between mannosylated PTP1 and an, as yet, unknown host cell mannose-binding molecule [35] that results in the adherence of the polar tube to its host cell [35–40]. PTP2 is found at the same genomic locus as PTP1; and the PTP2 proteins from various microsporidia, despite a high degree of sequence divergence, share common characteristics such as a basic isoelectric point, high lysine content and conservation of cysteine residues [29, 37, 41, 42]. Immunoscreening of an *E*. *cuniculi* cDNA library led to the identification of a third polar tube protein, PTP3 [30]. PTP3, along with PTP1 and PTP2, was also found in cross-linked polar tube complexes and these three PTPs have been demonstrated to interact in yeast two hybrid assays [30, 39]. It has been suggested that PTP3 may act as a scaffolding protein for the assembly of other PTPs during the developmental formation of the polar tube [30]. PTP4 and PTP5 were identified in a survey of *E*. *cuniculi* proteins, but their function and interactions with other PTPs are unknown [10, 43]. Similar to the PTP1/PTP2 gene cluster, the genes for PTP4 and PTP5 usually are in a cluster in the genome [10, 43]. Despite the identification of these various PTPs, we still do not understand how the polar tube is formed or the mechanisms utilized for host cell infection and attachment.

Herein, we report on our characterization of *Encephalitozoon hellem* PTP4 and the identification of Transferrin receptor 1 (TfR1) as a candidate host cell receptor for this polar tube protein. The interaction between these molecules is demonstrated to be important for cell infection by this microsporidia. This is the first report of a host cell receptor that is involved in binding of a polar tube protein and infection of host cells. A deeper understanding of the mechanisms of infection and the formation of the microsporidian invasion synapse should provide new therapeutic targets for management of these ubiquitous intracellular pathogens.

## Results

### Identification of EhPTP4 as a novel polar tube protein

**Fig 1:**
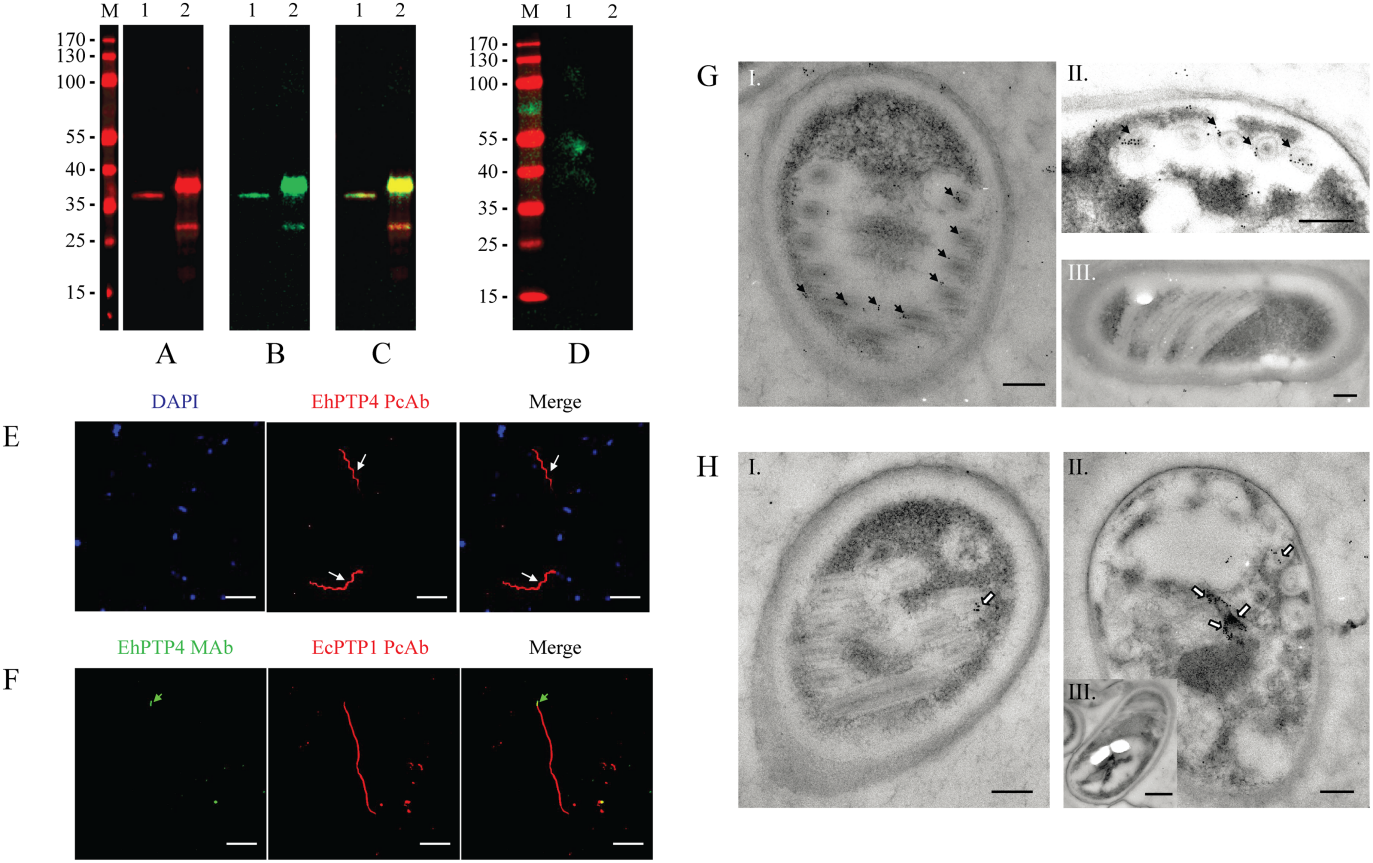
Characterization of *Encephalitozoon hellem* polar tube protein 4 (EhPTP4). **(A-C)** Immunoblot using (A) rabbit anti-EhPTP4 polyclonal antibody (rab-PcAb-EhPTP4) and (B) anti-EhPTP4 MAb (MAb-EhPTP4). (C) Merged image of (A) and (B), note that both rab-PcAb-EhPTP4 and MAb-EhPTP4 interact with the same protein in *E*. *hellem* spore lysate (lane 1) and with purified recEhPTP4 protein (lane 2). Donkey anti-mouse IRDye 800CW and donkey anti-rabbit IRDye 680RD were used as secondary antibodies. **(D)** Immunoblot with pre-immune rabbit serum and negative IgG mAb (control antisera). No bands were recognized by either of these control (negative) sera. Lane 1: *E*. *hellem* spore lysate; lane 2: purified recEhPTP4 protein. Donkey anti-mouse IRDye 800CW and donkey anti-rabbit IRDye 680RD were used as secondary antibodies. **(E)** Extruded polar tubes of *E*. *hellem* were initially incubated with rab-PcAb-EhPTP4 and detected by anti-rabbit Alexa Fluor 594 secondary antibody (red). White arrows indicate the labeling of EhPTP4 by rab-PcAb-EhPTP4. Bar, 10 μm. **(F)** Extruded polar tubes of *E*. *hellem* were initially incubated with MAb-EhPTP4 (green secondary antibody) and rabbit polyclonal antibody to polar tube protein 1, i.e. rab-PcAb-EhPTP1 which stains the entire polar tube (red secondary antibody), the merged panel demonstrates that MAb-EhPTP4 labels the tip of polar tube (green arrows). Control preimmune sera did not display any reactivity in this assay. Bar, 10 μm. Nuclei were visualized by DAPI stain (blue). **(G)** *E*. *hellem* spores were initially incubated with rab-PcAb-EhPTP4 and/or MAb-EhPTP4 followed by secondary antibodies conjugated with 10-nm colloidal gold. Mature *E*. *hellem* spores stained with rab-PcAb-EhPTP4. Panel (I) and (II) demonstrate that gold particles localize to the polar tube (black arrows). Panel (III), negative control. Bars, 200 nm. **(H)** Mature *E*. *hellem* spore incubated with MAb-EhPTP4 panel (I) and (II) demonstrate that gold particles localize to a single region on polar tube adjacent to the posterior vacuole (white arrows). Panel (III), negative control. Bar, 200 nm (Panel H-I and H-II), 500nm (Panel H-III).

Recombinant EhPTP4, (recEhPTP4, S1 Fig) without its signal peptide, was expressed in *Escherichia coli* as a fusion protein as detailed in the Materials and Methods Section and this recEhPTP4 was used to immunize both mice and rabbits. An EhPTP4 mouse monoclonal antibody (clone F4-6; MAb-EhPTP4) was produced by screening a hybridoma library. Both the rabbit polyclonal antibody (rab-Pc-EhPTP4Ab) and mouse monoclonal antibody (MAb-EhPTP4) to EhPTP4 react to the same antigenic band in *E*. *hellem* spore lysates (Fig 1A–1D, S2 Fig). This reactive band has a molecular mass of ~36kDa, which is slightly larger than the predicted EhPTP4 molecular mass of 32kDa. This difference from predicted mass is probably due to post translational glycosylation of PTP4 that is predicted to occur based on in silico analysis of this protein (S1A Fig). Indirect immunofluorescence assay (IFA) and transmission electron microscopy (TEM) have been used to successfully assess the localization of microsporidian proteins [39, 44–46]. IFA using rab-PcAb-EhPTP4 demonstrated that the entire polar tube was labeled by this polyclonal serum (Fig 1E and S3 Fig), proving that EhPTP4 is a polar tube protein; however, MAb-EhPTP4 labeled only the tip of polar tube (Fig 1F and S3 Fig). The different localization patterns seen using the rab-Pc-EhPTP4Ab and MAb-EhPTP4 (S3 Fig) suggests that there is an EhPTP4 epitope which is only exposed at the tip of polar tube and that this epitope is specifically recognized by the MAb-EhPTP4. Immunoelectron microscopy (ImmunoEM) was utilized to examine the location of EhPTP4 in *E*. *hellem* spores (Fig 1G and 1H). Consistent with the labeling result using IFA, the gold particles were observed on the polar tube when using rab-PcAb-EhPTP4 (Fig 1G-I and 1G-II) and there was no staining by control rabbit serum (Fig 1G-III). Using MAb-EhPTP4 a more limited region of the polar tube was stained with gold (Fig 1H-I), with the majority of gold particles being found at an area where the polar tube appeared to be connected to the posterior vacuole (Fig 1H-II). This is consistent with previous models of eversion of the polar tube [10, 47] where the tip of the tube should be located near the membrane of the posterior vacuole prior to germination. There were no gold particles seen in the control panel using an unrelated isotype matched MAb (Fig 1H-III). These combined IFA and TEM observations confirm that EhPTP4 is a polar tube protein and the localization of MAb-EhPTP4 to the tip of the polar tube suggests that EhPTP4 may be involved in an interaction with a host cell protein during infection.

### Correlated electron microscopy of the *E*. *hellem* invasion synapse

**Fig 2:**
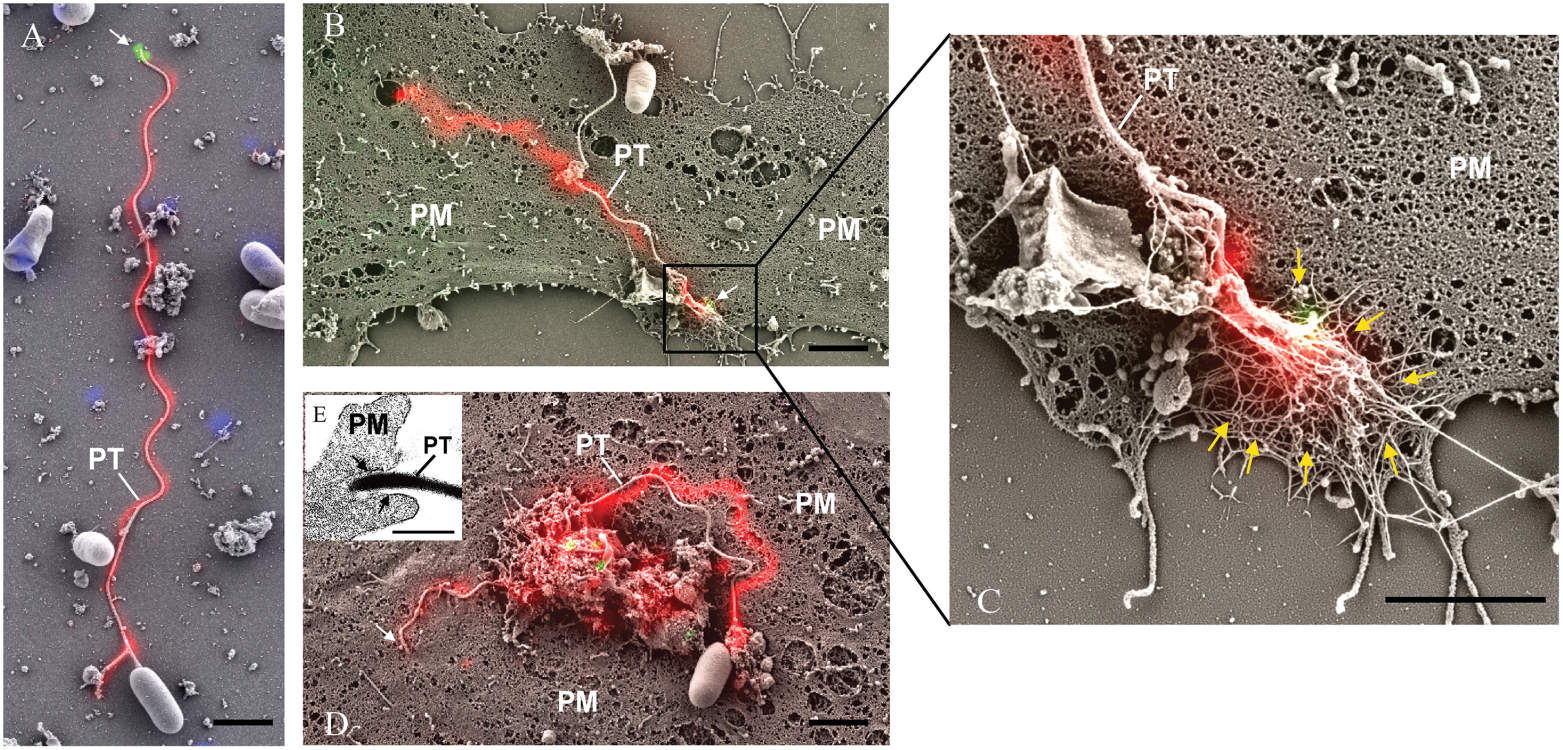
Correlative light and electron microscopy (CLEM) analysis of *E*. *hellem* infection. *E*. *hellem* infected host cells were initially incubated with rab-PcAb-EhPTP1 (rabbit polyclonal antibody; red) and MAb-EhPTP4 (mouse monoclonal antibody; green). The fluorescence image and SEM image of same sites were taken sequentially, and the fluorescence images of EhPTP4 and the polar tube were correlated to the SEM images. **Panel A** shows a germinated polar tube with the EhPTP4 staining at the end of tube (white arrow). **Panel B** shows the binding of polar tube to host cell plasma membrane during infection. **Panel C i**s an enlarged section of panel B, the tip of polar tube and some host fiber-like structures (yellow arrows). **Panel D** demonstrates penetration of polar tube into host cell plasma membrane, there was no EhPTP4 staining at the end of polar tube (white arrow), suggested that the end of polar tube had already entered the host cell and was below the level of imaging. **Panel E** shows TEM data demonstrating that the polar tube was surrounded with host cell membrane (black arrows) at the site of infection. PT, polar tube; PM, plasma membrane. Bar size: 2 μm panels A, B, C and D, and 400 nm panel E.

Correlated fluorescence and scanning electron microscopy (CLEM) combines wide-field laser/light microscopy with subsequent electron microscopy and can minimize the respective disadvantages of each technique when used individually [48, 49]. CLEM was used to investigate the polar tube during infection. Infected cells were fixed and stained with MAb-EhPTP4 (mouse monoclonal) and rab-PcAb-EhPTP1 (rabbit polyclonal) to locate EhPTP4 at the tip of the tube and EhPTP1 on the entire polar tube respectively. After being incubated with fluorescent secondary mouse and rabbit antibodies, a fluorescence microscopy image was taken, and then a scanning electron microscopy (SEM) image of the same site on the slide was taken. Finally, the fluorescence images that demonstrated localization of EhPTP4 and polar tube were correlated with the SEM images that demonstrated the invasion organelle of the microsporidia at a high resolution. As seen in Fig 2A, the germinated polar tube was labeled with rab-PcAb-EhPTP1 and MAb-EhPTP4 labeled the tip of polar tube, consistent with the results of the IFA and TEM studies. Fig 2B shows a discharged polar tube and the interaction of the polar tube tip with host cell plasma membrane (PM). As is shown in enlarged section (Fig 2C), the tip of polar tube is buried under some fiber-like structures of the host cell (these structures surround the invasion synapse). The mechanism by which the polar tube interacts with the host cell membrane resulting in penetration is currently unknown; however, there is some evidence that host cell actin may be involved in microsporidian penetration of the host cell within the invasion synapse [50]. The location of a specific epitope of EhPTP4 at the tip of the polar tube suggests that EhPTP4 may directly interact with host cell proteins/receptors in the final penetration of the host cell membrane during infection. After the binding of polar tube to host cell membrane, the next step in infection should be the initial penetration of polar tube into the host cell plasma membrane. Fig 2D demonstrates an apical tip of polar tube that has entered the host cell plasma membrane by pushing the host cell plasma membrane into the cell. Our TEM observations support the concept that the polar tube is surrounded by host cell membrane at the invasion site (Fig 2E). These observations are similar to previous reports regarding the mechanism of polar tube penetration in that the tip of polar tube is not piercing or breaking the host plasma membrane, but instead pushing the host cell plasma membrane into the host cell creating a microenvironment into which the microsporidian sporoplasm is extruded from the end of the polar tube [51–54].

### EhPTP4 binds to the host cell surface

**Fig 3:**
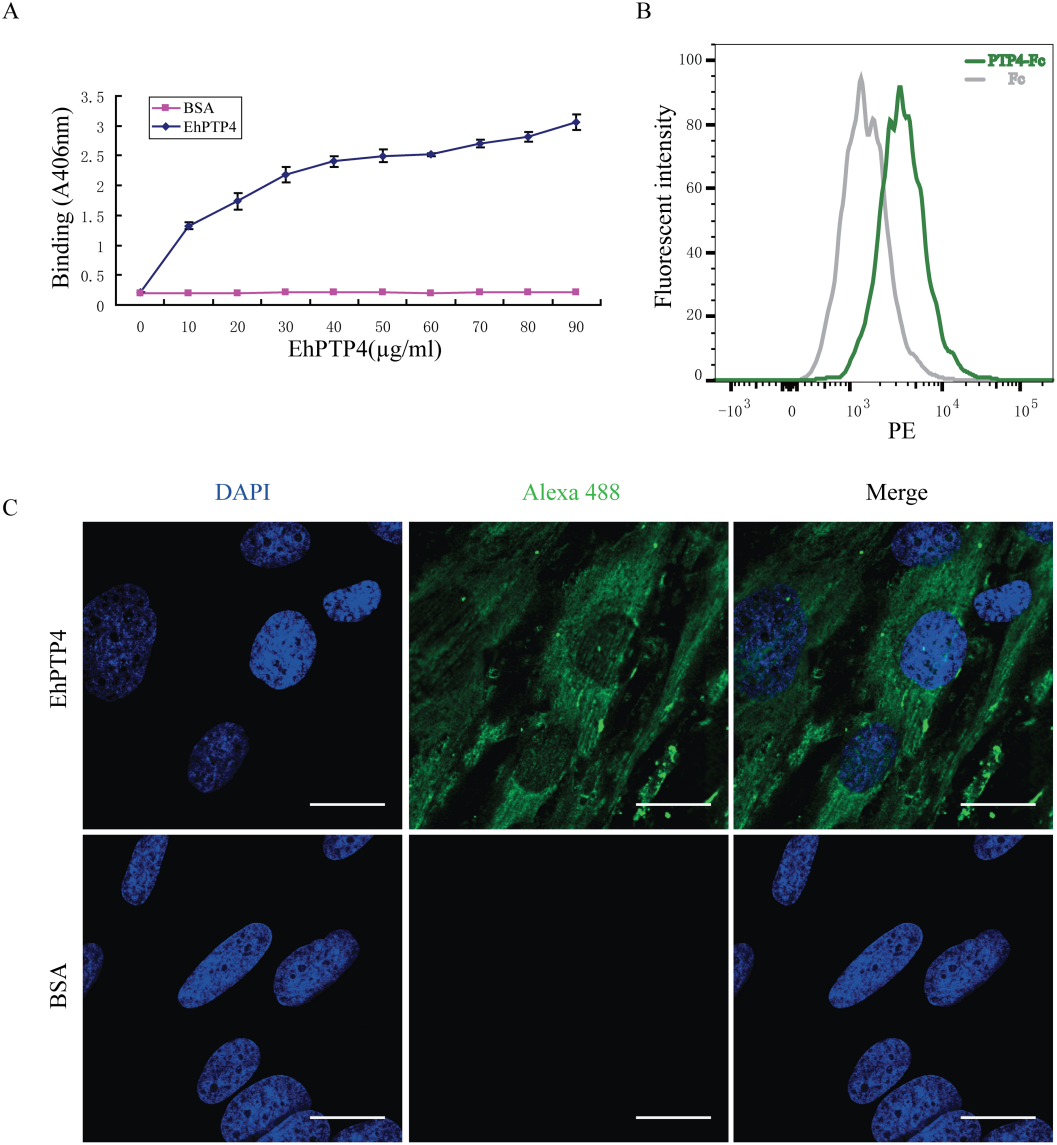
EhPTP4 binds to the surface of host cells. **(A)** ELISA detection of EhPTP4 binding to RK13 cells. Constructs with different concentrations were incubated with RK13 cells in 96 well plates and binding was detected via ELISA. BSA at the same concentration as EhPTP4 was used as a negative control for these assays. All experiments were performed in triplicate. Data shown are presented as mean +/- SD. **(B)** FACS detection of EhPTP4 binding to RK13 cells. RK13 cells were detached by treating with 1X Citric saline solution and incubated with EhPTP4-Fc fusion protein and human Fc protein. The observed shift in the FACS curve is consistent with EhPTP4 binding to RK13 cells. **(C)** Immunofluorescence microscopy (IFA) of EhPTP4 binding to RK13 cells. Constructs were incubated with RK13 cells grown on glass coverslips and binding was detected using fluorescent goat anti-mouse secondly antibody. BSA at the same concentration as EhPTP4 was used as a negative control for these assays. Bar, 20 μm.

These combined IFA, TEM and CLEM studies suggested that EhPTP4 may be involved in an interaction with the host cell membrane during infection. Binding of EhPTP4 to host cells was evaluated using ELISA, IFA and FACS (Fig 3). Increasing concentrations of recEhPTP4 were incubated with fixed RK13 cells on a 96 well plate and binding was detected as described in Materials and Methods. The results demonstrated that incubation of host cells with increasing amounts of EhPTP4 resulted in a proportional increase in binding (Fig 3A) suggesting that EhPTP4 most likely interacts with a protein (or other binding partner) on the host cell membrane; however, as the host cells used in these ELISA experiments were fixed, it is possible that EhPTP4 could have been binding to either an intracellular or extracellular binding site. To address this issue FACS analysis was done using live cells that were not fixed or permeabilized. FACS analysis (Fig 3B) demonstrated that EhPTP4 also bound to the cells and, as live cells were used, this binding was clearly on the host cell surface. RK13 cells were detached by citric saline solution and incubated with EhPTP4-Fc fusion proteins or human Fc proteins. After labeling with PE-conjugated anti-Fc antibody, binding was detected by flow cytometry. As demonstrated in Fig 3B, the binding curve of EhPTP4-Fc was significantly shifted compared to the control human Fc binding curve, confirming that EhPTP4 binds to host cells. When trypsin was used to remove surface proteins from HFF cells, there was no binding of EhPTP4 to host cells (S4A and S4B Fig). When recEhPTP4 was incubated with these RK13 cells followed by IFA the binding signal was clearly visualized on the host cell surface (Fig 3C). In summary, the binding data are consistent with an ability of EhPTP4 to interact with protein(s) on the host cell surface.

### Identification of Transferrin receptor 1 as a host cell receptor for PTP4

**Fig 4:**
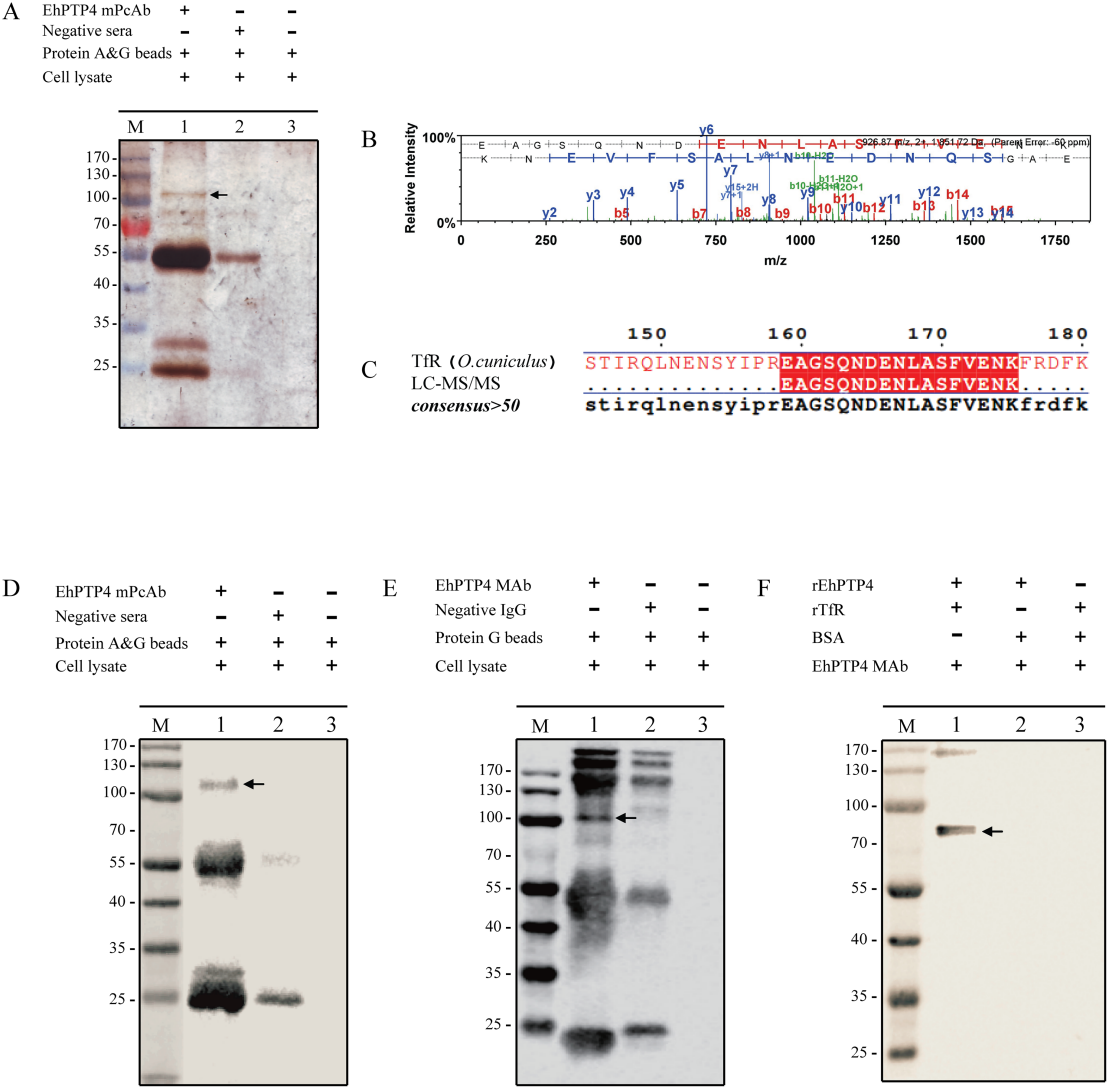
Transferrin receptor 1 (TfR-1) is a host cell interacting protein (HCIP) for EhPTP4. **(A)** Silver staining of immunoprecipitation (IP) samples precipitated by mouse anti-EhPTP4 polyclonal antibody (mo-PcAb-EhPTP4). Mouse pre-immune serum was used as negative control. Arrowhead indicates the band of interest in the IP. **(B, C)** The MS/MS spectrum of the peptide EAGSQNDENLASFVENK with spectral assignments matched well with the B and Y product ions which deduced the peptide sequence (B). A BLAST search using NCBI nr database found this peptide sequence to be unique to Transferrin receptor protein 1 (XP_002716583.1) from *Oryctolagus cuniculus* (rabbit host cell line) with an expectation E-value of 3e-08 and 100% sequence identity (C). **(D)** Immunoblot detection of TfR1 in samples precipitated by mo-PcAb-EhPTP4. Mouse pre-immune serum was used as a negative control. An arrowhead indicates the TfR-1 band identified by the immunoprecipitation assay. **(E)** Immunoblot detection of TfR1 in samples precipitated by MAb-EhPTP4. Mouse serum (mouse IgG) was used as a negative control. An arrowhead indicates the TfR-1 band identified by the immunoprecipitation assay. **(F)** Pull down assay of TfR-1 from a protein mixture of recEhPTP4 and recTfR-1 using MAb-EhPTP4 conjugated protein G sepharose beads. The protein samples were loaded on the SDS-PAGE gel, then transferred to a PVDF membrane and probed with anti-TfR1 PcAb. An arrowhead indicates the band corresponding to recTfR-1 (77.4 kDa).

Immunoprecipitation (IP) was utilized to identify the potential interacting targets of EhPTP4 on its host cell. RK13 (rabbit) cells were infected with *E*. *hellem* spores and harvested after two weeks of infection. The lysate of infected cells was incubated with protein A&G sepharose beads that had been conjugated with mouse polyclonal antibody to EhPTP4 (mo-PcAb-EhPTP4). The proteins that bound to the beads, i.e. the immunoprecipitation sample (IP sample), were then analyzed by SDS-PAGE. Silver staining demonstrated that there were several bands which were unique to the IP sample (lane 1) compared to the control samples (lane 2 and 3) (Fig 4A), and an *Oryctolagus cuniculus* (rabbit) protein, Transferrin receptor 1 (TfR1) was identified by LC-ESI-MS/MS analysis (Fig 4B and 4C). The same band was also identified using a TfR1 monoclonal antibody (MAb-TfR1; see details in Materials and methods section) (Fig 4D). We repeated the IP using MAb-EhPTP4 (monoclonal anti-PTP4) and the same TfR1 band was identified in this IP using MAb-TfR1 as shown in Fig 4E. Furthermore, pull down assays using antibody to EhPTP4 (MAbEhPTP4) demonstrated that recEhPTP4 can directly interact with recTfR-1 *in vitro* (Fig 4F and S5 Fig).

**Fig 5:**
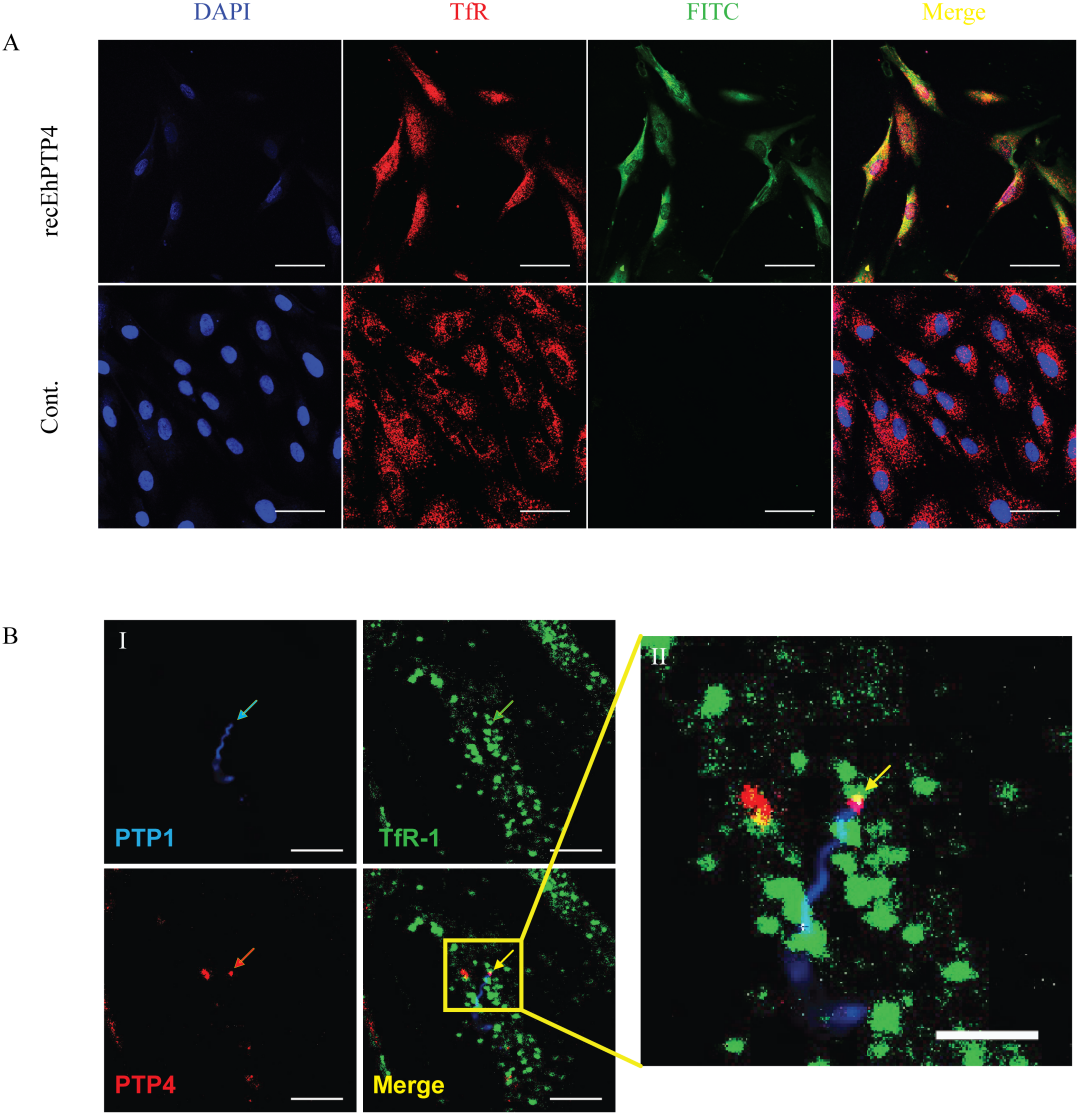
Confocal laser scanning IFA co-localization analyses of EhPTP4 and TfR-1 on the surface of HFF cells. **(A)** Recombinant EhPTP4 was incubated with uninfected HFF cells grown on glass coverslips. After fixation and blocking the HFF cells were incubated with MAb-EhPTP4 (green) and Anti-TfR-1 (red) as described in Materials and Methods. The merged image demonstrates localization of EhPTP4 and TfR-1 on the HFF. Bar, 50 μm. **(B) B-I** shows *E*. *hellem* infected HFF cells that were fixed and incubated with rab-Pc-EhPTP1 (blue), TfR-1 MAb (green) and MAb-EhPTP4 (red). The color coded arrows indicate staining by each antibody. The final panel in **B-I** shows the overlay of the polar tube (blue), the tip of polar tube (red) and TfR-1 (green). Bar, 10 μm. **B-II** is an enlarged section of the B-I overlay image demonstrating (yellow arrow) the juxtaposition (yellow) of the tip of the polar tube (red) and TfR-1 (green). Bar, 5 μm. As a negative control for recombinant EhPTPT4 the same amount of BSA was used in place of EhPTP4 and no staining was observed. In addition, control antisera (preimmune rabbit serum and isotype matched monoclonal antibodies) did not stain cells.

IFA co-localization techniques using EhPTP4 Alexa Fluor 488 (green) and TfR1 Alexa Fluor 594 (red) staining on host cells demonstrated signal at the site of infection for both EhPTP4 and TfR1 (Fig 5A). During microsporidian infection we could also find that the tip of the polar tube co-localized with TfR1 on host cell membrane at the site of the invasion synapse (Fig 5B). Therefore, several lines of evidence suggest that TfR1 is a host cell receptor that can interact with EhPTP4. We next examined if this interaction had an effect on the infection process.

### Inhibition of EhPTP4 binding inhibits microsporidia infection

**Fig 6:**
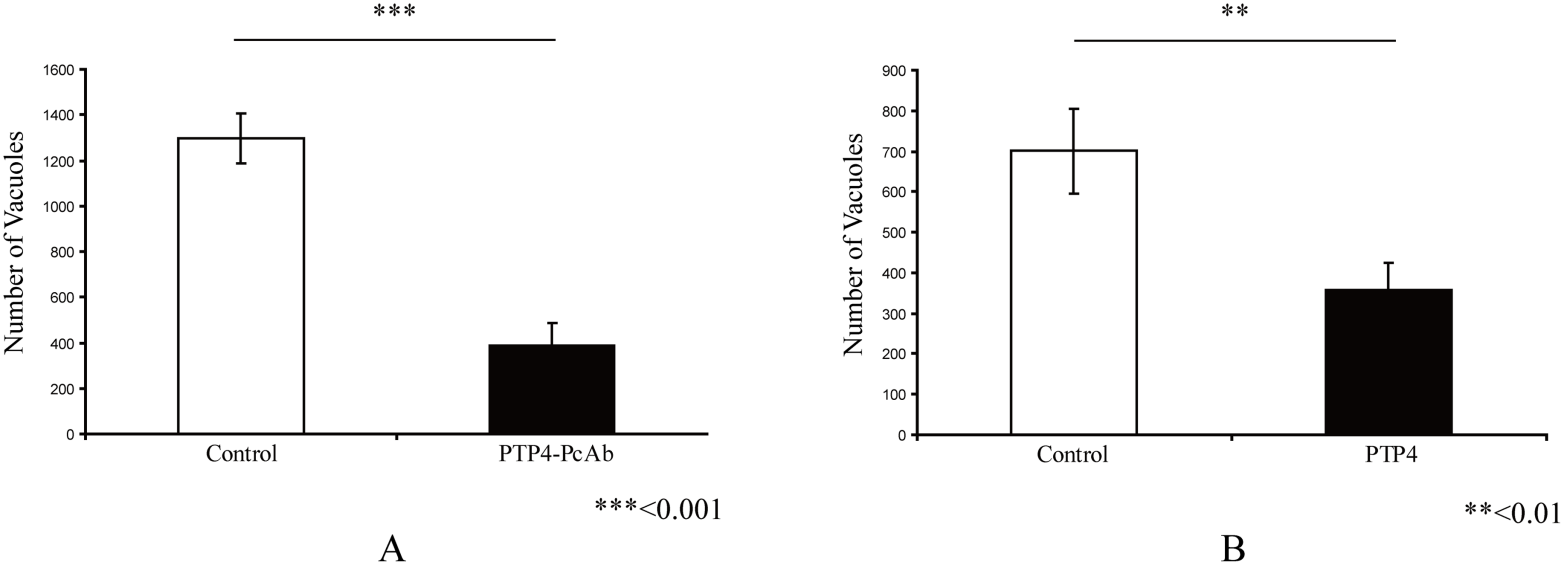
Interference of EhPTP4 host cell interactions alters infectivity. **(A)** Impact of rabbit polyclonal anti-EhPTP4 (rab-PcAb-EhPTP4) on *E*. *hellem* infection of HFF cells. *E*. *hellem* spores were incubated with rab-PcAb-EhPTP4 (~5 μg/ml) for 1hr at room temperature and then this mixture was added to HFF monolayers, the same amount (~5 μg/ml) of seronegative rabbit serum was used as negative control to assess the specificity of the effect of rab-PcAb-EhPTP4 serum. As can be seen rab-PcAb-EhPTP4 significantly reduced the number of HFF cells infected by *E*. *hellem*. **(B)** Impact of exogenous recPTP4 on *E*. *hellem* infection of HFF cells. Purified recEhPTP4 (5 μg/ml) was incubated with HFF cells for 1hr at room temperature, the HFF were then infected with 1×10^6^ spores/well, the infected HFFs were incubated for 9 days at 37°C 5% CO_2_, and then stained and infection rates were calculated. The same amount of BSA (5μg/ml) was used as negative control. As can be seen recEhPTP4 significantly reduced the number of HFF cells infected by *E*. *hellem*.

To further examine the role of EhPTP4 during microsporidia infection (see S6 Fig for assay details), we performed antibody blocking and protein competition experiments. EhPTP4 rabbit polyclonal antibody (rab-PcAb-PTP4) was incubated with purified spores for 1hr before infecting host cells. We found a significant reduction in infectivity as a result of blocking EhPTP4 interactions with rab-PcAb-PTP4 in the antibody blocking assay (Fig 6A). To perform a protein competition assay, recombinant EhPTP4 protein was directly added into cell culture prior to infection with spores and the number of parasitophorous vacuoles was counted after 9 days post-infection. Similar to what was seen with rab-PcAb-PTP4, adding EhPTP4 recombinant protein to cell culture during infection significantly decreased the infectivity of microsporidia (Fig 6B). Taken together, these data indicate that PTP4 plays a role in infection.

### Alterations of Transferrin receptor 1 (TfR1) interactions inhibits microsporidia infection

**Fig 7:**
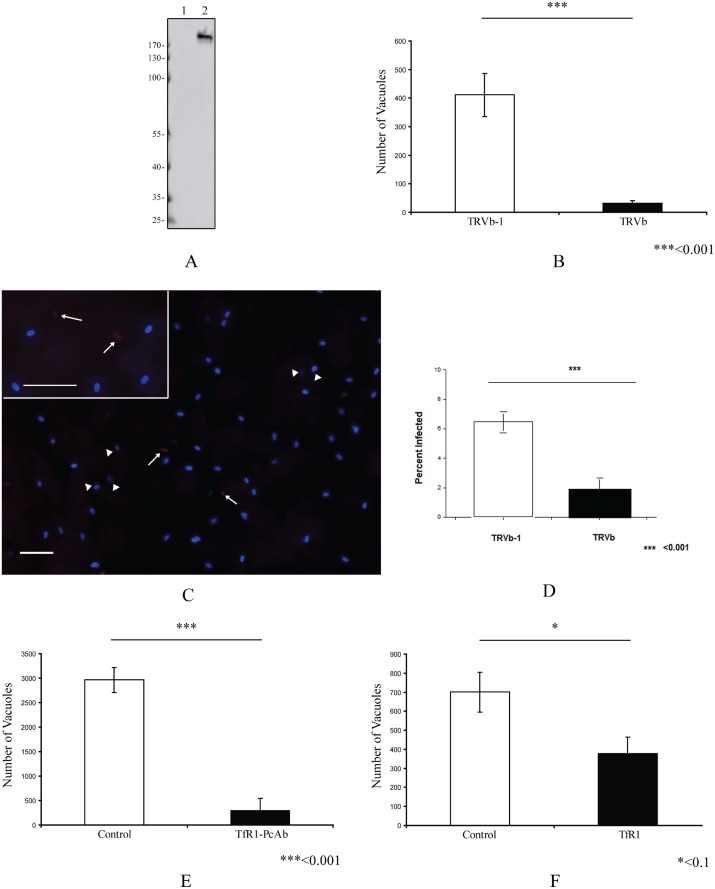
Effect of the manipulation of host cell TfR1 expression on *E*. *hellem* infection. **(A)** Immunoblot of TRVb cells (lane 1) and TRVb-1 cells (lane 2) using MAb-TfR1. **(B)** The CHO cell lines TRVb (a TfR1 knockout) and TRVb-1 (that expresses human TfR1) were infected with *E*. *hellem* and infection rates determined as described in Materials and Methods. A significant decrease in infection was observed in TRVb-1 cells compared to TRVb cells, which is consistent with binding to TfR1 being involved in the infection process. **(C**) Fluorescent microscopy (using a triple band D/F/TR XF467 Omega Optical filter cube and a 40X objective; insert is a 60X objective) of *E*. *hellem* invasion assay at 6 hours post infection. *E*. *hellem* infected CHO cell lines, TRVb or TRVb-1, were incubated for 6 hours with spores labeled with Calcofluor white, the cells were then fixed and *in situ* hybridization with an *E*. *hellem* rRNA probe labeled with Alexa Fluor 594 was performed to distinguish intracellular forms (red fluorescence at white arrows) from extracellular adherent spores (blue spores) as described in Materials in Methods. The white arrowheads point to spores, that we believe, are in the process of invading where the sporoplasm is at the edge of the spore, but where intracellular invasion has not yet been completed or occurred. Bars, 10μm. **(D)** Percent of CHO cell lines TRVb or TRVb-1 infected at 6 hours. A significant decrease (p<0.001) in invasion (red intracellular forms) was observed in TRVb (TfR1 knockout) cells compared to TRVb-1 (expressing human TfR1) cells. Data are presented as mean +/- SEM. **(E)** Effect of rabbit anti-TfR polyclonal antibody (rab-PcAb-TfR1) on *E*. *hellem* infection of HFF cells. HFF cells were pre-incubated with 5 μg/ml rab-PcAb-TfR1 or seronegative rabbit serum for 1hr, HFF were then infected by 1×10^6^ *E*. *hellem* spores/well and infection assayed as described in Materials and Methods. A significant decrease in infection was observed in HFF treated with rab-PcAb-TfR1 **(F)** Effect of exogenous recTfR1 on infection. Following an overnight culture HFF cells incubated with human TfR1 or BSA 5 μg/ml per well (24 well plate) for 1hr at room temperature, the HFFs were then infected with 1×10^6^ spores/well, incubated for 9 days and infection measured as described in the Materials and Methods section. A significant decrease in infection was observed in HFF treated with human TfR1.

To further evaluate if TfR1 plays a role in infection, we evaluated the effect of alternations of TfR1 expression in host cells on the ability of microsporidia to infect these cells. The CHO cell lines TRVb, which is a TfR1 knockout, and TRVb-1, which expresses human TfR1, were used to examine microsporidia infection. Immunoblot analysis using MAb-TfR1 confirmed that there is no TfR1 protein expressed in the TRVb cell line (lane 1, Fig 7A), and that TfR1 is expressed in the TRVb-1 cell line (lane 2, Fig 7A). Microsporidian infection was dramatically decreased in TRVb cells compared to the infection rate seen in TRVb-1 cells (Fig 7B). In order to examine the role of TfR1 in invasion we evaluated the number of intracellular *E*. *hellem* organisms 6 hours after infection (Fig 7C). Microsporidia invasion was significantly decreased in TRVb cells compared to TRVb-1 cells (Fig 7D). In order to further examine the role of TfR1 in infection, an antibody blocking assay and TfR1 competition assay were performed as described in the Materials and Methods section of this paper. Analogous to the infection results in TRVb cells, we also observed a dramatically decreased infection rate in cells whose TfR1 was blocked by polyclonal antibody to TfR1 (Fig 7E). Furthermore the addition of recombinant TfR1 protein (recTfR1) into cell culture during microsporidia infection also decreased host cell infection rates (Fig 7F). This blocking of infection by recTfR1 may be a consequence of the binding of recTfR1 to PTP4 preventing the interaction of PTP4 with native host cell TfR1, thereby, interfering with downstream events due to this interaction. In summary, these data suggest that host cell TfR1 is involved in the microsporidian infection process.

## Discussion

Microsporidia possess a unique invasion apparatus, the polar tube. Under the appropriate environmental stimulation, the polar tube can discharge rapidly out of microsporidia spore, form a hollow tube and serve as a conduit for the passage of sporoplasm and nuclear material into a new host cell [51]. Before germination, within the spore, the polar tube is filled with material that has been hypothesized to consist of unpolymerized polar tube proteins and during the process of germination and extrusion of the polar tube this filled structure transitions to a hollow tube [23, 55]. The length of discharged polar tubes is approximately 2 to 3 times that of the coiled tubes inside the spore and it has been hypothesized that either unpolymerized polar tube proteins are incorporated at the growing tip of polar tube during discharge or that the tube unfolds during eversion [23, 27, 28, 55]. In the process of polar tube eversion, unique immunologic epitopes may be exposed on polar tube proteins [10].

In the current study we have provided a characterization of PTP4 and identified a role for this protein in infection. Both monoclonal and polyclonal antibodies to EhPTP4 recognize the same band in spore lysates by immunoblotting, suggesting that these antisera recognize the same protein and that any difference in staining patterns is not due to recognition of additional proteins by the polyclonal antiserum. A monoclonal antibody to PTP4 (MAb-EhPTP4; clone F4-6) was found to specifically label the anterior end of polar tube while the polyclonal rabbit antibody to EhPTP4 (PcAb-EhPTP4) labeled the entire polar tube. This is consistent with the exposure of a specific epitope on EhPTP4 at the tip of the polar tube that is recognized by this monoclonal antibody, and which defines the location of the tip of the polar tube. Since the tip of polar tube is the place where polar tube sporoplasm exits and the location of an interaction with the host cell membrane in the microenvironment of the invasion synapse, these localization data suggest that PTP4 may be involved in polar tube host-cell interactions in the invasion synapse and in the mechanism of penetration of the host cell by microsporidia. Analyses employing IFA, ELISA and FACS confirmed that PTP4 binds mammalian cells. When EhPTP4 binding was inhibited by antibody treatment of host cells microsporidia infection was reduced (Fig 6A), suggesting that EhPTP4 interaction with the host cell is involved in the infection process.

Despite the identification of several polar tube proteins in the last few years [29–31], it is not known how the PTPs or the polar tube interact with host cells or how penetration occurs. In this manuscript, we provide evidence that TfR1, a host cell membrane protein, interacts with PTP4 and that this interaction is important in the process of infection. Proteomic analysis of PTP4 bound to host cell membranes purified by co-IP identified Transferrin receptor 1 (TfR1) as a potential host cell interacting partner for PTP4. Data obtained by immunoprecipitation, pull down and immunocolocalization provide additional confirmation of an interaction between EhPTP4 and TfR1. Knockout of TfR1 in CHO cells significantly decreased the infectivity of microsporidia. Antibody blocking of TfR1 or the addition of recombinant TfR1 protein to cell culture also reduces microsporidia infectivity.

The Transferrin receptor 1 (TfR1) is involved in iron uptake in many cells. Cells can import iron by internalizing the transferrin-iron complex through clathrin-mediated endocytosis [56]. TfR1 binds iron-loaded transferrin at a neutral pH, and releases the iron in the early acidic endosome and then recycles back to the cell surface [57]. TfR1 is the cell receptor for a variety of viruses including parvoviruses, hepatitis C virus, mammary tumor virus and arenaviruses, and this receptor can be utilized by those virus to bind, invade and infect host cells [58–62].

Interestingly recEhPTP4 was found to bind to both TRVb CHO and TRVb-1 CHO cells (S4C Fig) suggesting that EhPTP4 may bind to host cells by several mechanisms. According to our analysis of the protein sequence of EhPTP4 (S1B Fig), there are several carbohydrate binding domains predicted to occur consistent with previously reported structures of chitin-binding proteins [63]. Both Cys87 and Gly90 of EhPTP4 are conserved amino acid residues that are involved in binding chitin in previously described proteins (S1C Fig), indicating that EhPTP4 might be able to bind chitin. Therefore, a possible explanation for recEhPTP4 binding to both TRVb CHO and TRVb-1 CHO cells is that the chitin domain may allow EhPTP4 to bind to glycoproteins on host cells (or facilitate other receptor interactions). Binding to these alternative sites would explain the ability of recEhPTP4 to bind to TRVb cells. Despite this, it is clear that the presence of functional TfR1 is important in the infection process and that EhPTP4 binding to TfR1 is involved in *E*. *hellem* host cell infection. The data also suggest a role for TfR1 in the invasion synapse in addition to any role TfR1 may have in the binding/adherence of the polar tube to the host cell surface.

**Fig 8:**
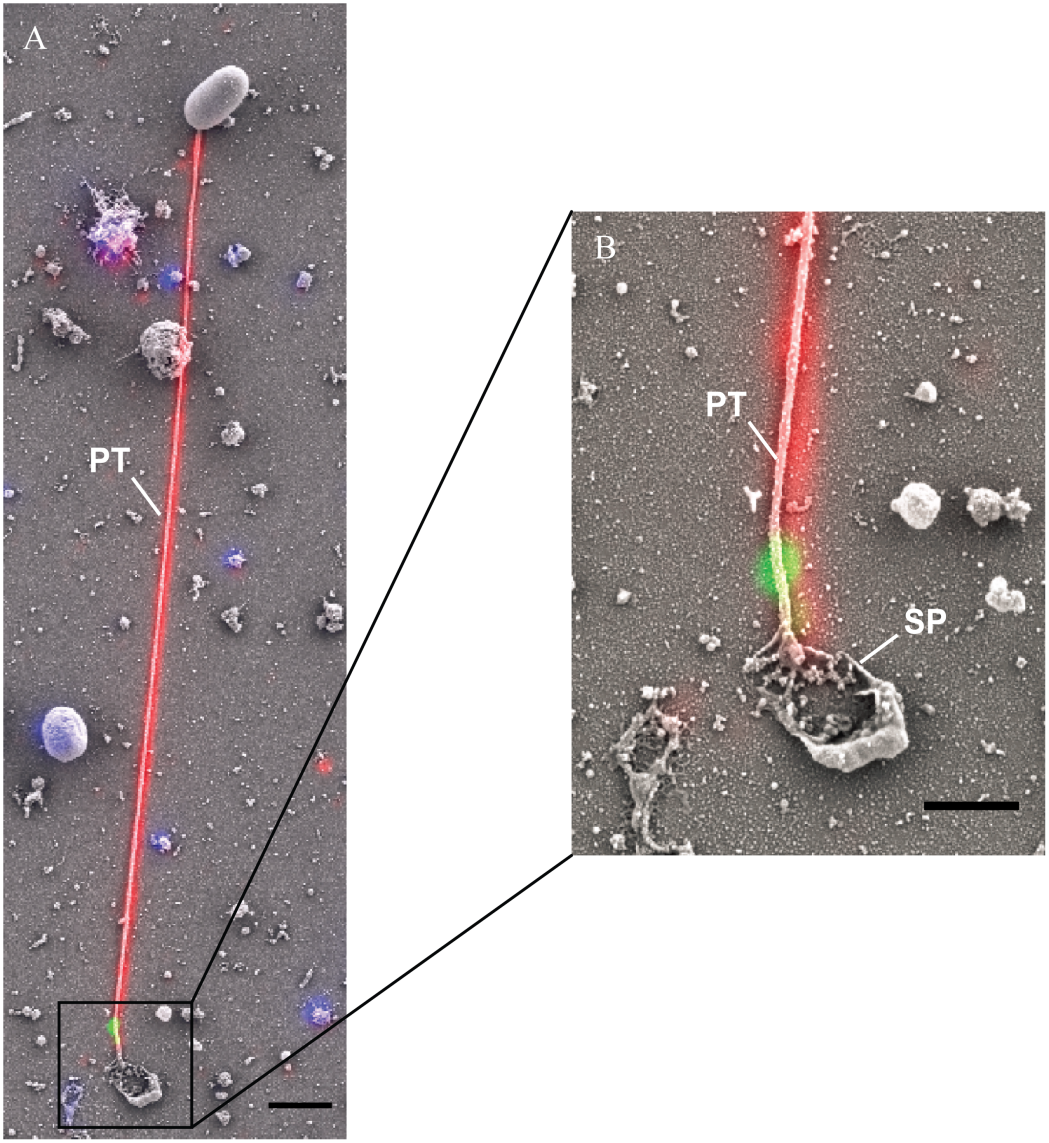
Correlative light and electron microscopy (CLEM) analysis of *E*. *hellem* germination. *E*. *hellem* infected host culture were initially incubated with rab-PcAb-EhPTP1 (red) and MAb-EhPTP4 (green). The fluorescence image and SEM image of same site were taken sequentially, and the fluorescence images showing the labeling of EhPTP4 and the polar tube were correlated to the SEM images which demonstrated the germination of a microsporidium at high resolution. Panel (A) shows an extruded polar tube with EhPTP4 staining at the end of tube. Bar, 2 μm. Panel (B) shows the enlarged section of panel (A), the droplet of released sporoplasm (SP) was still attaching to the tip of polar tube. Bar, 1 μm. PT, polar tube. SP, sporoplasm.

It has been suggested that the polar tube is extracytoplasmic in the unfired spore [64]. After the discharge of the polar tube is completed, the sporoplasm will flow through the polar tube and appear as a droplet at the distal end [26, 65]. If a spore discharges next to a host cell, it will pierce the cell membrane and transport sporoplasm into the cell [26, 27, 65]. If there are no cells in the area, the droplet will remain attaching to the end of polar tube for a period of time (Fig 8). CLEM clearly demonstrates the PTP4 is located at the place where the polar tube connects with droplet spore contents (Fig 8). As is shown in Fig 1H-II, the end of polar tube which was labeled with EhPTP4 MAb was connected with posterior vacuole area, suggesting that the localization of the end of polar tube in spore is probably next to the posterior vacuole in the microsporidian spore.

**Fig 9:**
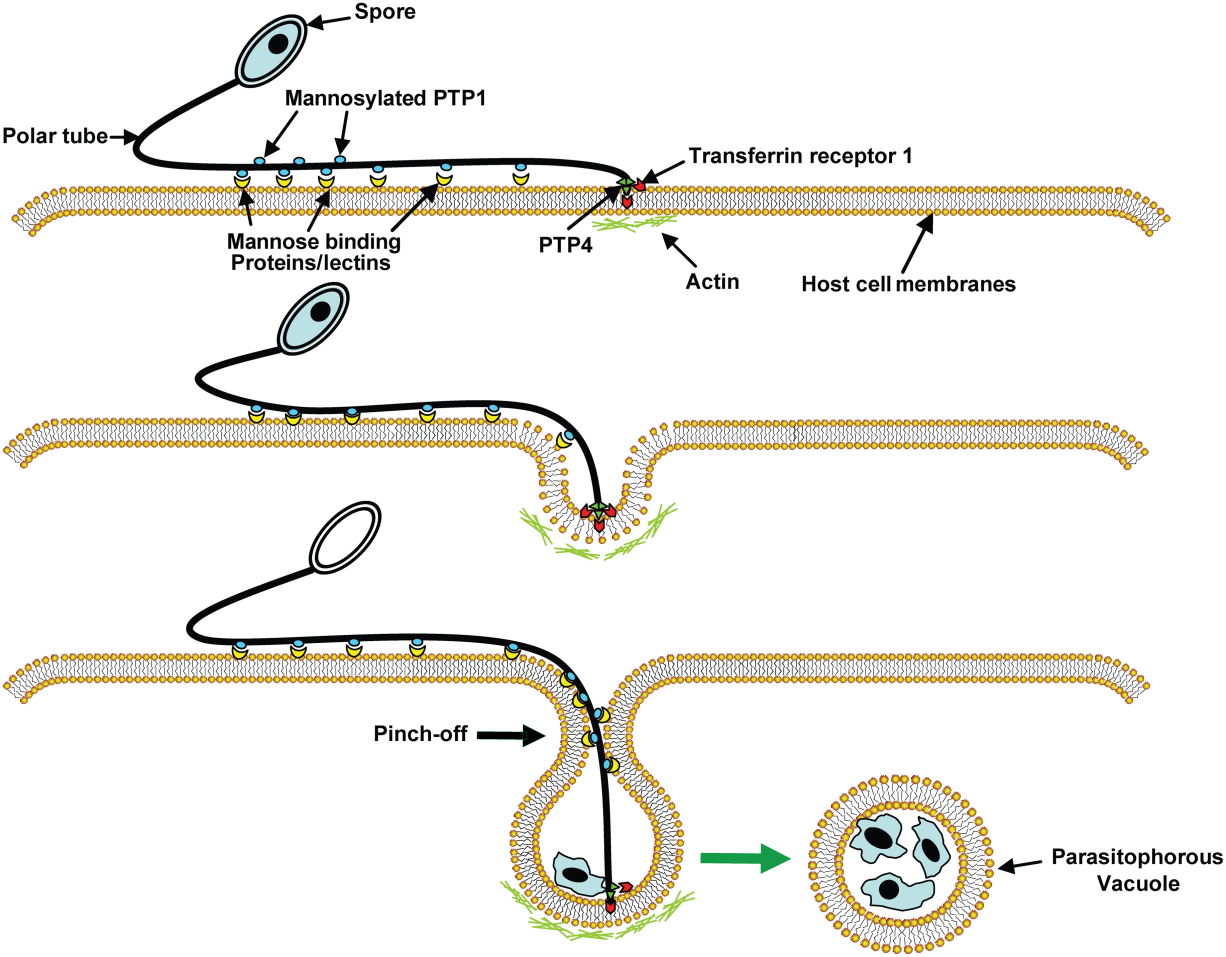
A model of microsporidian host cell infection. Polar tube protein 1 (PTP1) interacts with mannose binding proteins (MBP) on the host surface adhering the polar tube to the host surface allowing the polar tube to form an invasion synapse by pushing into the host cell membrane. Interactions of PTP1 (and possibly PTP4) with the host cell membrane in the invasion synapse exclude the external environment from the invasion synapse creating a protected microenvironment for the extruded microsporidian sporoplasm. Polar tube protein 4 (PTP4) epitopes at the tip of polar tube interact with Transferrin receptor 1 (TfR1) or other host cell interacting proteins (HCIPs) on the host cell surface triggering signaling events such as clathrin-mediated endocytosis and the involvement of host cell actin in the final invasion event with formation of a parasitophorous vacuole.

On the basis of previous reports [34, 50, 51] and the current data, we have proposed an infection model for microsporidia (Fig 9). According to this model, upon appropriate environment stimulation the polar tube rapidly discharges out of spore and then polar tube protein 1 (PTP1), which was shown to be a mannosylglycoprotein, interacts with mannose binding proteins or lectins on host cell surface [34, 35] allowing the polar tube to adhere to the host cell surface. PTP4 interactions, with TfR1 or with glycoproteins, may also facilitate adherence of the polar tube to the host cell surface. As the polar tube everts it pushes into the host cell creating a protective microenvironment, the invasion synapse, into which the sporoplasm is then extruded. Interactions with PTP1 (and perhaps PTP4) with the host cell membrane during the formation of this invasion synapse allow the creation of an invasion synapse microenvironment that is to be isolated from the external environment. PTP4 epitopes that are exposed at the tip of polar tube can interact with TfR1 in the protected environment of the invasion synapse. This interaction probably triggers clathrin-mediated endocytosis pathways helping to facilitate the progress of infection [51, 66]. When the extruded sporoplasm is injected into the invasion synapse the vacuole membrane will pinch off from host cell membrane and form a parasitophorous vacuole (PV) and any associated TfR1 will be lost from PV membrane immediately after infection being recycled in the cell [67]. Observations by other laboratories suggest that host cell actin may have also has a role in the final invasion event [50]. The precise mechanisms of the EhPTP4 interaction with TfR1, how this complex functions and dissociates in the PV after infection and whether PTP4 interacts with other proteins on the host cells remains an unknown and important area for further investigation.

## Materials and methods

### Cells and reagents

RK13 (rabbit kidney) cells (ATCC, CCL-37) and CHO cells (TRVb and TRVb-1) (gift of Dr. Colin R. Parrish and Dr. Timothy E. McGraw, Cornell University) were cultured in 10% fetal bovine serum (FBS) (ThermoFisher) Minimum Essential Medium Eagle (MEM) with penicillin-streptomycin at 5% CO2. Human foreskin fibroblasts (HFF) (ATCC, CRL-2522) and 293FT (ATCC, CRL-3216; gift of Dr. Matthew Levy, Albert Einstein College of Medicine) cells were maintained in Dulbecco’s Modified Eagle Medium (DMEM; ThermoFisher Scientific) with penicillin-streptomycin (ThermoFisher Scientific) supplemented with 10% FBS (ThermoFisher Scientific) at 5% CO_2_. Anti-human TfR1 mouse monoclonal antibody (mo-Mab-HuTfR1; clone H68.4) used for immunoblot and IFA was purchased from Invitrogen. Anti-human TfR1 rabbit polyclonal antibody (rab-PcAb-TfR1) used for blocking and IFA was purchased from Santa Cruz Biotechnology. PE-conjugated anti-human Fc antibody (clone HP6017) used for flow cytometry was purchased from Sony Biotechnology. The lipofectamine 3000 transfection kit used for 293FT cell transfection was purchased from Invitrogen. HRP-conjugated anti-mouse secondary antibodies, Alexa Fluor 488 conjugated goat anti-mouse secondary antibody and Alexa Fluor 594 conjugated goat anti-rabbit secondary antibody were purchased from ThermoFisher Scientific. Rabbit anti-EhPTP1 polyclonal antibody (rab-PcAb-EhPTP1) used for IFA and CLEM was produced previously in our laboratory [33]. Buffered solutions and chemicals were purchased from Sigma Aldrich. All reagents used are commercially available and chemicals were of the highest analytical grade available from the supplier.

### *Encephalitozoon hellem* culture

RK13 (rabbit kidney) cells were maintained in 10% fetal bovine serum MEM with penicillin-streptomycin at 5% CO_2_. Confluent monolayers were infected with *E*. *hellem* spores. The spores were collected from culture media, purified by passing them through 5 μm size filter (Millipore, Billerica, MA) to remove host cells, concentrated by centrifugation, and stored in sterile distilled water at 4°C. Spores used in these experiments were counted with a hemocytometer (three times/sample and averaged).

### Cloning, protein expression and purification of EhPTP4

The coding region of EhPTP4 was PCR amplified from *E*. *hellem* genomic DNA using Q5 High-Fidelity DNA Polymerase (New England Biolabs) with specific primers (S1 Table) and ligated into the vector pMCSG7 containing a TEV cleavable N-terminal hexahistidine (His6) tag. The resulting plasmid was transformed into BL21 (DE3) T1R (Sigma-Aldrich) containing the RIL plasmid (RIL) from Stratagene (CA, USA) containing copies of genes encoding tRNAs for rare codon. Transformed bacteria were grown in Luria Broth (LB; Sigma-Aldrich) containing ampicillin and chloramphenicol (100 μg/μl and 34 μg/μl, respectively), for 5hrs at 37°C. At that time the temperature was lowered to 22°C for overnight growth. The bacterial cells were pelleted and resuspended in buffer A (20 mM Hepes pH 7.6, 500 mM NaCl, 20 mM imidazole, 10% glycerol, 0.01% Tween-20, 0.1% NaN_3_, containing 1 mM PMSF and 4 u/ml of DNAase I). The cells were lysed using an EmulsiFlex C3 (Avestin) and separation of the lysate from the intact cells was achieved by centrifugation (16500 g, 1hr). The pellet was washed two times in buffer B (20 mM Hepes pH 7.6, 150 mM NaCl, 0.1% Triton X100, 10 mM DTT) and another time in buffer B without Triton X100. The pellet was then solubilized in buffer C (100 mM NaH_2_PO_4_, 10 mM Tris, 6 M guanidine at pH 8.0). The supernatant was saved after centrifugation (16500Xg, 1hr) and loaded onto a His-Pure Ni-NTA column (ThermoFisher Scientific) that had been previously equilibrated with buffer C. The column was successively washed with 10 column volumes of buffer D (100 mM NaH_2_PO_4_, 10 mM Tris, 8 M urea, 2% Triton X100 at pH 8.0), followed by 5 column volumes of buffer D without Triton X100, and then 5 column volumes of buffer E (100 mM NaH_2_PO_4_, 10 mM Tris, 8 M urea, 2% Triton X100 at pH 6.3). The protein was then eluted with 5 column volumes of buffer F (100 mM NaH_2_PO_4_, 10 mM Tris, 8 M urea, 2% Triton X100 at pH 4.5). Protein purity was determined by SDS-PAGE and protein concentration determined using a nanodrop spectrophotometer (ThermoFisher Scientific).

### Expression of EhPTP4-Fc fusion protein

The coding region of EhPTP4 was PCR amplified using Q5 High-Fidelity DNA Polymerase (New England Biolabs) employing EhPTP4 specific primers (S1 Table), ligated into vector pFUSE-hIgG1-Fc2 (a gift from Dr. Matthew Levy), and then transfected into 293FT cells using Lipofectamine 3000 Reagent (Thermo Fisher Scientific). A mock transfection was also prepared with the empty vector pFUSE-hIgG1-Fc2. 293FT cells grown to 70% ~90% confluency in six well plate were prepared in 3 ml fresh medium (Dulbecco’s modified Eagle medium with 10% FBS and 1%). The transfected reagent was prepared by diluting 2.5 μg plasmid with 250 μl Opti-MEM medium and 10 μl P3000 reagent, followed by adding additional diluted Lipofectamine 3000 regent. The solution was mixed well and incubated for 5 min at room temperature before adding to the cells. Supernatant containing EhPTP4-Fc fusion protein or Fc protein was harvested 48hrs later after transfection.

### Antibody production

For polyclonal antibody production in mice 500 μg of purified recEhPTP4 protein was injected into five BALB/c mice after emulsification of the protein with Freund's complete adjuvant (Sigma-Aldrich) at a dose of 100 μg protein for each mouse. After boosting, using 100 μg protein per mouse in Freund’s incomplete adjuvant (Sigma-Aldrich) monthly for three months, the mice were bled and serum (mo-PcAb-EhPTP4) stored at -20°C until use. Control mouse sera was collected from BALB/c mice prior to immunization (pre-immune serum) as well as BALB/c mice of the same age which had not been immunized with recEhPTP4 (i.e. control polyclonal mouse IgG).

Polyclonal rabbit antibody to recEhPTP4 was produced by Harlan Laboratories (Envigo, USA) following their standard 112 day polyclonal antibody protocol. Briefly, a New Zealand white rabbit was initially immunized with 200 μg of purified recEhPTP4 emulsified with Freund’s complete adjuvant and this was then followed by three monthly injections of 100 μg of purified recEhPTP4 emulsified with Freund’s incomplete adjuvant. The ELISA titer of the rabbit serum was assessed following each boost injection and was over 1:50,000 one month after the final injection. Rabbit serum (PcAb-EhPTP4) was collected one month following the final immunization and stored at -20°C. Prior to immunization serum was collected and screened to confirm that this rabbit did have any endogenous antibody that reacted with *E*. *hellem* or EhPTP4. Preimmunization rabbit serum stored -20°C was used as a negative control for experiments using polyclonal rabbit antisera.

For monoclonal antibody production, the spleen cells of mice which were immunized with recEhPTP4 (100 μg protein per mouse) were fused with a myeloma cell line to create hybridoma libraries according to our previously protocol [68]. An ELISA was used to screen the resultant hybridoma libraries. For the ELISA, 0.05 μg per well recEhPTP4 was coated onto 96 well plates, and then the plates were blocked with 3% BSA in TBST for 1hr. The hybridoma cell culture media were then incubated with the EhPTP4 coated plates, and hybridomas that were ELISA positive were then picked and grown in 6 well plates. The selected hybridomas were then screened again by immunoblot using recEhPTP4 and total spore lyses as antigens. The monoclonal lines that corresponded to a single band on immunoblot were then screened by IFA using germinated spores. Hybridoma cells that stained polar tubes in IFA were chosen and then subcloned on agarose and rescreened by ELISA and IFA. The monoclonal hybridoma cell line of interest, clone F4-6 (isotype IgG1), was then cultured in CELLine bioreactor (Integra Biosceinces) for large-scale production of MAb-EhPTP4.

### Transmission electron microscopy (TEM)

RK13 cells were cultured in six well plates to confluence and infected with 1×10^6^ spores for 3 days. Cells were fixed with 4% paraformaldehyde 0.05% glutaraldehyde in 0.1M sodium cacodylate buffer, dehydrated through a graded series of ethanol, with a progressive lowering of the temperature to -50°C in a Leica EMAFS, embedded in Lowicryl HM-20 monostep resin (Electron Microscopy Sciences), and then polymerized using UV light as we have previously published [26]. Ultrathin sections were cut on a Reichert Ultracut E, immunolabeled with antibodies of interest and then stained with uranyl acetate (Electron Microscopy Sciences) followed by lead citrate. Stained sections were viewed on a JOEL 1200EX transmission electron microscope at 80kv [33].

### Correlated fluorescence and scanning electron microscopy (CLEM)

1×10^5^ RK13 cells were infected with 1×10^6^ spores on glass coverslips imprinted with three location markers. Cells were fixed with 4% paraformaldehyde in PBS for 30 min after three days post-infection. After being washed three times by TBST (TBS plus 0.05%Tween 20), coverslips were blocked by 3% BSA in TBST for 1hr at room temperature. EhPTP4 monoclonal antibody (MAb-EhPTP4; clone F4-6) at a 1:10 dilution and EhPTP1 rabbit polyclonal antibody (rab-PcAb-EhPTP1) at a 1:500 dilution were incubated with cells for 1hr at room temperature. After being washed three times by TBST, Alexa Fluor 488-labeled anti-mouse IgG antibody and Alexa Fluor 594-labeled anti-rabbit IgG antibody were added at 1:500 dilutions. Mouse and rabbit preimmunization sera were used as negative controls. Samples were washed three times by TBST and imaged using a Zeiss AxioObserver microscope equipped with Axiovision software with "shuttle & find" to mark cell locations. After fluorescence imaging, samples were fixed with 2.5% glutaraldehyde, dehydrated in ethanol, critical point dried (Tousimis Samdri 790), and coated with chromium (EMS 150T-ES). The same cells were automatically located in the Zeiss Supra 40 Field Emission Scanning Electron Microscope and imaged with a secondary electron detector. Fluorescence and SEM images were correlated with Zeiss AxioVision.

### ELISA binding assay

RK13 cells were grown to confluence in 96-well plate and fixed with 4% paraformaldehyde in PBS for 30 min at room temperature. Non-specific binding sites were blocked by incubating with 3% BSA in TBST buffer. After washing three times with TBST, serial diluted EhPTP4 soluble protein (starting at 100 μg/ml) was added to each well and incubated 1hr at room temperature. BSA at the same concentration as EhPTP4 was used as negative control for all ELISA assays. Unbounded proteins were washed off by washing three times with TBST, and bound proteins were fixed by incubating with methanol for 10 min. After washing with TBST for three times, non-specific binding sites were blocked by incubating plates with 3% BSA in TBST for 1hr at room temperature, EhPTP4 mouse polyclonal sera (mo-PcAb-EhPTP4) was then added at 1:500 dilution, plates were incubated for 1hr at room temperature and then washed three times with TBST. After this wash anti-mouse IgG alkaline phosphatase antibody (Sigma-Aldrich) was added at a 1:7000 dilution, plates were incubated for 1hr at room temperature, washed three times with TBST, and then p-nitrophenyl phosphate substrate (Sigma-Aldrich) was added and absorbance at 405 nm was read using a MRXe microplate reader (DYNEX Technologies). BSA at the same serial dilution was used as negative control.

### Cell binding flow cytometry assay

EhPTP4-Fc fusion proteins used for flow cytometry were produced from 293t cells transfected with the appropriated plasmids. HFF cells were cultured to confluence and detached by treating with 1X Citric saline (135 mM KCl, 15mM sodium citrate, diluted with sterile distilled water) solution for 5 min at 37°C. The detached cells were washed with PBS containing 1% bovine serum albumin (FACS buffer). 1×10^5^ cells were allowed to incubate with the supernatants harvested from 293t cell culture for 1hr. After the cells were washed twice with FACS buffer, PE anti-human IgG Fc (Sony Biotechnology) was added to each sample and allowed to incubate for 30 min. Followed incubation with DAPI for 10 min to identify dead cells, which were removed from the FACS analysis, flow cytometry was performed on a Becton Dickinson LSRII Analyzer Flow Cytometers (BD Biosciences).

### Immunofluorescence assay (IFA)

To detect the binding of EhPTP4 by IFA, RK13 cells were grown to confluence in four-well chamber slides and fixed by 4% paraformaldehyde in PBS for 30 min at room temperature. After washing three times with TBST, slides were blocked by 3% BSA in TBST for 1hr at room temperature, and 500 μl 20 μg/ml EhPTP4 recombinant protein or BSA was added and incubated for 1hr at room temperature. Unbound proteins were then removed by washing three times with TBST, and bound proteins were fixed with methanol for 10 min. Non-specific binding sites were blocked by incubation with 3% BSA in TBST and EhPTP4 polyclonal sera (mo-PcAb-EhPTP4) was added at a 1:500 dilution in 3% BSA in TBST. After incubating for 1hr at room temperature, slides were washed three times with TBST, Alexa Fluor 488-labeled anti-mouse IgG antibody was added at a 1:500 dilution in 3% BSA in TBST, and then the slide was incubated for 1hr at room temperature. After washing three times with TBST, slides were mounted with ProLong Gold antifade regent and photomicrographs were taken either with a SP5 confocal microscope (Leica) or Microphoto-FXA epifluorescence microscope (Nikon).

To detect the localization of EhPTP4 on microsporidia spores, infected RK13 cells were grown on four-well chamber slides (ThermoFisher Scientific) and fixed with 4% paraformaldehyde in PBS for 30 min. After washing three times with TBST, slides were blocked by 3% BSA in TBST for 1hr at room temperature. Anti-EhPTP4 monoclonal antibody (MAb-EhPTP4) and EhPTP1 rabbit polyclonal antibody (rab-PcAb-EhPTP1 [33]) were added at 1:10 and 1:500 dilutions respectively in 3% BSA in TBST and incubated 1hr at room temperature. After washing three times with TBST, Alexa Fluor 488-labeled anti-mouse IgG antibody and Alexa Fluor 595-labeled anti-rabbit IgG antibody were added at a 1:500 dilution. After washing three times with TBST, slides were mounted with ProLong Gold antifade regent and viewed with a SP5 confocal microscope (Leica). Mouse preimmunization serum was used as a negative control.

### Immunoprecipitation and LC-MS/MS analysis

RK13 cells were cultured in a T75 flask (Corning) to confluence and then infected with 1×10^7^ spores. The cells were harvested two weeks post-infection and cell lysates were obtained by a modified procedure as reported previously [34]. Infected RK13 cells were spun down and disrupted in 1% SDS lysis buffer containing protease inhibitor (Protease inhibitor cocktail; Thermo Fisher Scientific) with 0.4 g 0.5 μm acid-washed glass beads (Sigma-Aldrich) for 1 min on a Mini-Beadbeater (BioSpec Products). The disrupted host cell suspension was then clarified by centrifugation at 12000 rpm for 10 min.

For immunoprecipitation, 50 μl of Protein A&G agarose beads in PBS was incubated with 20μl of mo-PcAb-EhPTP4 or mouse pre-immune serum at 4°C for 1hr. The protein A&G agarose beads conjugates were washed three times with PBS and resuspended in a final volume of 500 μl cell lyses collected above. The samples were incubated at 4°C for 2hrs with gentle shaking. The samples were then centrifuged, and the pellets were washed three times with PBS. Fifty μl of 2X protein sample buffer (0.5M Tris-HCl pH 6.8, 4.4% SDS, 20% glycerol, 2% 2-mercaptoethanol and 0.01% bromophenol blue) was added to each sample and the samples were then boiled for 5 min. Following centrifugation the supernatant was loaded onto an SDS-PAGE gel and electrophoresed. The gel was then stained with Coomassie Brilliant Blue or silver (using a BioRad Silver staining kit) and analyzed using methods that we have previously reported [68]. Briefly, excised gel bands were reduced, alkylated and digested with trypsin. LC-ESI-MS/MS (liquid chromatography electrospray ionization mass spectrometry) analysis of the peptide digests was then done by C18-Reversed Phase (RP) chromatography using an Ultimate 3000 RSLCnano System (ThermoFisher Scientific) equipped with an Acclaim PepMap RSLC C18 column (2 μm, 100 Å, 75 μm x 15 cm, ThermoScientific, USA). The UPLC was connected to a TriVersa NanoMate nanoelectrospray source (Advion) and a linear ion trap LTQ-XL (ThermoFisher Scientific) mass spectrometer with ESI source operated in the positive ionization mode. MGF files generated from the raw LC-ESI-MS/MS data were searched by Mascot (version 2.5.1, Matrix Science, USA) against NCBInr90_20141124 database (25,782,812 protein sequences) with the following search parameters: trypsin; two missed cleavages; peptide charges of +2 and +3; peptide tolerance of 1.5 Da; MS/MS tolerance of 0.5 Da; carbamidomethylation (Cys) for fixed modification; deamidation (Asn and Gln) and oxidation (Met) for variable modifications. An appropriate decoy database search was utilized to measure the false discovery rate.

The immunoprecipitation was also repeated using with MAb-EhPTP4, briefly 50 μl of Protein G sepharose beads in PBS was incubated with 20 μl of MAb-EhPTP4 or mouse negative IgG at 4°C for 1hr. The protein G sepharose beads conjugates were washed three times with PBS and resuspended in a final volume of 500 μl cell lyses collected above. Samples were incubated at 4°C for 2hrs with gentle shaking, then centrifuged, and the pellets were washed three times with PBS. Fifty μl of 2X protein sample buffer was ere added to each sample and the samples were then boiled for 5 min. The IP samples were then precipitated by using either mo-PcAb-EhPTP4 or MAb-EhPTP4, were run on SDS-PAGE, and then transferred to a PVDF membrane. Immunoblot analysis was then performed using MAb-TfR1 to evaluate the presence of TfR1 in the IP material.

### Pull-down assay to examine an interaction of recTfR-1 with recEhPTP4

For the pull down assay, 20 μg recTfR-1 and 20 μg recEhPTP4 were incubated at 4°C for 3 hrs in PBS with gentle shaking. Fifty μl of Protein G sepharose beads in PBS were incubated with 20 μl of MAb-EhPTP4 or 20μg of BSA (as a negative control) at 4°C for 1hr with gentle shaking. The protein G sepharose beads conjugates were washed three times with PBS and then incubated with the protein mixture at 4°C for 2hrs with gentle shaking. The samples were then centrifuged, the pellets were washed three times with PBS and 50 μl of 2X protein sample buffer (0.5M Tris-HCl pH 6.8, 4.4% SDS, 20% glycerol, 2% 2-mercaptoethanol and 0.01% bromophenol blue) was added to each sample. Samples were then boiled for 5 min, subjected to SDS-PAGE electrophoresis, transferred to a PVDF membrane and examined by immunoblot using PcAb-TfR1.

### Fluorescence co-localization assay

For co-localization of recombinant EhPTP4 with Transferrin receptor 1, human foreskin fibroblasts (HFF) cells were seeded in 4 well chamber slides and fixed with 4% paraformaldehyde. After being blocked with blocking buffer (3% BSA in TBST), HFF cells were incubated with 5 μg/ml of recombinant EhPTP4 or BSA for 1hr at room temperature. After being washed three times with TBST, anti-human Transferrin receptor 1 rabbit polyclonal antibody and anti-EhPTP4 mouse monoclonal antibody (MAb-EhPTP4) were incubated with HFF cells for 1hr at the dilution of 1:50 and 1:100 respectively. After washing three times with TBST, Alexa Fluor 488-labeled anti-mouse IgG antibody and Alexa Fluor 594-labeled anti-rabbit IgG antibody were added at a 1:500 dilution, and incubated for 1hr. After washing three times with TBST, slides were mounted with ProLong Gold antifade regent and viewed with a SP5 confocal microscope (Leica).

For co-localization of the tip of polar tube with TfR1, human foreskin fibroblasts (HFF) cells were seeded in 4 well chamber slides and infected with *E*. *hellem*. Three days post-infection the cells were fixed and blocked with blocking buffer. After being washed three times with TBST, anti-EhPTP4 mouse monoclonal antibody (MAb-EhPTP4) and anti-EhPTP1 rabbit polyclonal antibody (rab-Pc-EhPTP1) were incubated with these infected HFF cells for 1hr at dilutions of 1:100 and 1:500 respectively. After washing three times with TBST, Alexa Fluor 594-labeled anti-mouse IgG antibody and Alexa Fluor 405-labeled anti-rabbit IgG antibody were added at a 1:500 dilution, and slides were incubated for 1hr. After washing three times with TBST the slides were fixed with methanol for 10 min, rinsed once in TBST to remove methanol and then incubated with FITC conjugated anti-human TfR1 mouse monoclonal antibody (Fisher scientific) for 30 min at 4°C, protected the slides from light. After washing three times with TBST the slides were mounted with ProLong Gold antifade regent and viewed with a SP5 confocal microscope (Leica).

### Infectivity competition and blocking assay

To further analyze the roles of PTP4 and TfR1 in spore infection a protein inhibition assay and an antibody blocking assay were designed. The HFF cell line was used in these assays.

For the protein competition assay, 1×10^5^ HFF cells were cultured in 24 well plates overnight. Purified EhPTP4, TfR1, or BSA (as a negative control) was added to HFF cells at 5 μg/ml and then cells were incubated for 1 hr at room temperature. 1×10^6^ spores were used to infect the HFF monolayers which were then incubated for 9 days until infection was evident. Cells were fixed with 4% paraformaldehyde for 30 min at room temperature. After being washed with PBS for three times, 0.01% Calcofluor White (Sigma-Aldrich) was added into each well and stained for 10 min at room temperature and the parasitophorous vacuole number (infection rate) of each well were counted using Microphoto-FXA epifluorescence microscope (Nikon).

For the EhPTP4 antibody blocking assay, 1×10^5^ HFF cells were cultured in 24 well plates overnight. Anti-PTP4 rabbit polyclonal antibody (rab-PcAb-EhPTP4) or Rabbit seronegative (negative control for polyclonal rabbit antiserum) at a concentration of 5 μg/ml was incubated with 1×10^6^ spores for 1hr at room temperature. Then the antibody and spores mixture were added to the HFF monolayers and cells were incubated for nine days until the number of parasitophorous vacuole were counted as described above.

For the TfR antibody blocking assay, 1×10^5^ HFF cells were cultured in 24 well plates overnight. Anti-HumanTfR rabbit polyclonal antibody (rab-PcAb-HuTfR) or Rabbit seronegative (negative control for polyclonal rabbit antiserum) at a concentration of 5 μg/ml was added to HFF cells and the cells were incubated for 1hr at room temperature. 1×10^6^ spores were added to the HFF monolayers and cells were incubated for nine days until the number of parasitophorous vacuole were counted as described above.

### Infection of *E*. *hellem* in TRVb cells and TRVb-1 CHO cell lines

In order to examine the role of TfR1 during the infection of host cells by microsporidia, TRVb cells which do not express endogenous TfR and TRVb-1 cells which stably express the human TfR were utilized (these CHO cell lines were the kind gift of Drs. Parrish and McGraw, Cornell University) [64]. 1×10^5^ TRVb or TRVb-1 cells were cultured in six well plates and infected with 1×10^6^ spores. Fresh media was added every three days until the cells were fixed by 4% paraformaldehyde two weeks post-infection. Samples were washed with PBS followed by staining with 0.01% Calcofluor white (Sigma, USA) for 10 min at room temperature and the parasitophorous vacuole number (infection rate) of each well were counted using Microphoto-FXA epifluorescence microscope (Nikon).

### *E*. *hellem* invasion assay

E. hellem spores were purified from the culture supernatant of infected RK13 cells as described above by passage through a 27 gauge needle, followed by a 5 μm Nucleopore filter (Millipore, Billerica, MA) to remove host cells, concentrated by centrifugation, then stained with 0.01% Calcofluor White (Fluorescent Brightener 28; Sigma) for 10 min at room temperature, and then washed 3 times with PBS. These spores were immediately used for infection of CHO cells that had been grown on coverslips in 24 well plates. CHO cells, TRVb-1 and TRVb, were seeded at 1 x10^5^ cells per well into 24 well plates with each well containing a round glass coverslip. The cells were grown overnight to 90% confluence and then infected with 10^6^ labeled spores per well (an MOI of ~4:1). After incubation/infection for 6 hours, the coverslips in the 24 well plates were washed gently with PBS to remove free spores, fixed with 1:1 methanol:acetone for 10 minutes at room temperature, the fixative was then removed and the coverslips were allowed to be air dry at room temperature for 30 minutes. In situ hybridization with an *E*. *hellem* Alexa 594-oligo-RNA probe was performed using published methods [69, 70]. Prehybridization of the coverslips was performed with 100μl hybridization buffer (Sigma-catalogue number 11717472001) for 1 hour at 57°C in a humid chamber. Hybridization was then carried out by removing the prehybridization solution and then adding 20uM of the HEL878F probe [70], Alexa Fluor 594-ACTCTCACACTCACTTCAG (ThermoFisher Scientific, HPLC purified custom oligonucleotide), in hybridization buffer (Sigma) at 57°C overnight on a circular rocking platform in a humid chamber. The coverslips were then washed twice with 2X SSC for 15 minutes at 57°C and then mounted with ProLong Gold Antifade Mountant with DAPI (ThermoFisher Scientific). Coverslips were evaluated using Nikon Microphot FXA Microscope employing a triple band D/F/TR filter cube (XF467; Omega Optical, Brattleboro, VT) to determine infectivity rates. Twenty high power (40X) fields (HPFs) were counted for red (intracellular) forms and for the number of CHO cells in each HPF to determine the percent invasion at 6 hours post infection. Only red intracellular forms were counted as having invaded. Any spores that had both red and blue staining were not counted as these were interpreted as representing spores that were either very early in invasion or not able to correctly invade the host cells. Blue spores were not counted as these were interpreted as being extracellular spores adherent to the host cells. Invasion rates were expressed as percent invasion ± SEM and tested for statistical significance as described below.

### Ethics statement

All animal experiments were conducted according to the U.S.A. Public Health Service Policy on Humane Care and Use of Laboratory Animals. Animals were maintained in an AAALAC-approved facility and all protocols were approved by the Institutional Care Committee of the Albert Einstein College of Medicine, Bronx, New York (Animal Protocols 20150903, 20150904 and 20150905; Animal Welfare Assurance number A3312-01).

No human samples were used in these experiments. Human foreskin fibroblasts were obtained from ATCC.

### Statistics

All of the experiments above were performed in triplicate and repeated at least three times. Each experiment was performed separately with its own negative control. The significance of differences between control and experimental assays was evaluated using two-tailed Student’s T test employing R software for analysis. *P* values of 0.05 or less were considered statistically significant; *P* values of 0.01 or less were considered highly significant. Data was also analyzed using the Mann-Whitney U test (nonparametric statistics) and this confirmed the same degree of significance seen with the two-tailed Student’s T test.

### Nucleotide sequence accession number

The sequence of *E*. *hellem* PTP4 has been deposited in the GenBank database under accession number EHEL_071080.

## Supporting information

S1 Text Supporting information. (DOCX)

S1 Fig Protein sequence analysis of EhPTP4. **(A)** Diagram of protein sequence of EhPTP4. A carbohydrate binding domain (chitin binding domain and cellulose binding domain) predicted by SMART (http://smart.embl-heidelberg.de/) are indicated. Highly conserved residues among PTP4 homologs are shown in red. N-glycosylation sites and O-glycosylation sites are shown in green. A signal peptide predicted in EhPTP4 is shown in blue. **(B)** Multiple-sequence alignment of EhPTP4 and homologs. Four cysteine residues were highly conserved among PTP4 homologous (red triangles). The N-terminal of PTP4 homologs was relatively highly conserved compared to the C-terminal. **(C)** Chitin binding domain analysis of PTP4. Cys, Pro, and Gly have significant influence on the structure constructions of chitin-binding proteins. Cys^87^ and Gly^90^ of EhPTP4 are conserved among known chitin binding proteins (red stars), revealed that EhPTP4 may be also a chitin binding protein. Tachycitin: *T*. *tridentatus* tachycitin. Pj-CHIT1: *Penaeus japonica* chitinase 1. Peritrophin: 44-kDa glycoprotein from *Lucilia cuprina*. Ch-chit1: *Chelonus sp*. Chitinase. Ag-chit: *Anopheles gambiae* chitinase. Tn-IM: *Trichoplusia ni intestinal* mucin. WGA-A: *Wheat germ* agglutinin. Ac-AMP2: *Amaranthus caudatus* antimicrobial protein 2. Hevein: Hevein from *rubber tree*.
(TIF)

S2 Fig Immunoblot detection of native EhPTP4 in spore lysate immunoprecipitated by either MAb-EhPTP4 or PcAb-EhPTP4. **(A)** Mouse serum (preimmunization) was used as negative control. Arrowhead indicates the native EhPTP4 band pulled down by MAb-EhPTP4 and probed with rab-PcAb EhPTP4. **(B)** Rabbit serum (preimmunization) was used as negative control. Arrowhead indicates the native EhPTP4 band pulled down by rab-PcAb EhPTP4 and probed with MAb-Ab EhPTP4.
(TIF)

S3 Fig Immuno-localization of *Encephalitozoon hellem* polar tube protein 4 (EhPTP4). Extruded polar tubes of *E*. *hellem* were initially incubated with rab-PcAb-EhPTP4 and MAb-EhPTP4 detected by anti-rabbit Alexa Fluor 594 secondary antibody (red) and anti-mouse Alexa Fluor 488 secondary antibody (green). Blue arrows indicate the labeling of the nuclei of *E*. *hellem*; Red arrows indicate the labeling of the entire polar tube by rab-PcAb-EhPTP4; Green arrows indicate labeling of the tip of polar tube by MAb-EhPTP4; Yellow arrows indicate the merged signal of the rab-Pc EhPTP4 and MAb-EhPTP4 staining. Bar, 5 μm.
(TIF)

S4 Fig FACS detection of EhPTP4 binding to host cells. HFF cells were detached by treating with 1X Citric saline solution and incubated with EhPTP4-Fc fusion proteins or Fc proteins. The curve of EhPTP4 (Red) is significantly shifted compared to Fc curve (grey), revealed that EhPTP4 could bind to HFF cells **(A).** However, neither EhPTP4-Fc nor Fc protein could bind to the trypsinized cells on which the surface proteins were removed by trypsinization **(B)**. The transferrin receptor 1 (TfR1) knockout CHO cell line TRVb and a CHO cell line TRVb-1 which expresses human transferrin receptor 1 (huTfR1) were used to examine binding of EhPTP4 to these cells. EhPTP4 was able to bind to both TRVb or TRVb-1 cells **(C)**. This suggests that, in addition to TfR1, EhPTP4 can interact with other host cell proteins or post translational modifications on the host cell surface.
(TIF)

S5 Fig FACS detection of the direct interaction of TfR-1 and EhPTP4 in vitro. An immuno-pull-down assay was performed using MAb-EhPTP4 conjugated protein G sepharose beads which were then incubated with FITC conjugated anti-human TfR1 mouse monoclonal antibody. The red curve demonstrates that the pull down using recTfR-1 and recEhPTP4 mixtures was significantly shifted compared to the pull down result from recTfR-1 and BSA (green curve) or recEhPTP4 and BSA mixtures (blue curve). This result was consistent with the pull down results demonstrated by immunoblot in Fig 4F.
(TIF)

S6 Fig *E*. *hellem* Infection Kinetics in HFF cells. **(A)** Time dependent infection of *E*. *hellem* in HFF cells, mature spores could be identified starting at 3 days post-infection. The spore wall was stained with Calcofluor White (blue), cells were stained with GelRed (red). **(B)** TEM of a microsporidian parasitophorous vacuole (PV) in HFF cells at 6 days post-infection. **(C)** Time dependent *in vitro* growth curve of visible *E*. *hellem* PVs in HFF cells.
(TIF)

S1 Table List of primers used in this study. (DOC)

## References

[1] Sprague V (1977) Systematics of the Microsporidia Microsporidia Comparative Pathobiology 2 (Bulla L.A. Jr. & Cheng T.C.. Editors) Plenum Press pp. 31–334.

[2] Keeling PJ, Fast NM (2002) Microsporidia: biology and evolution of highly reduced intracellular parasites. Ann Rev Microbiol 56: 93–116. 12142484 10.1146/annurev.micro.56.012302.160854

[3] Sapir A, Dillman AR, Connon SA, Grupe BM, Ingels J, et al (2014) Microsporidia-nematode associations in methane seeps reveal basal fungal parasitism in the deep sea. Frontiers Microbiol 5. 10.3389/fmicb.2014.00043 PMC3918590 24575084

[4] Higes M, Martín R, Meana A (2006) *Nosema ceranae*, a new microsporidian parasite in honeybees in Europe. J Invert Path 92: 93–95. 10.1016/j.jip.2006.02.005 16574143

[5] Desportes I, Charpentier YL, Galian A, Bernard F, Cochand Priollet B, et al (1985) Occurrence of a new microsporidan: *Enterocytozoon bieneusi* ng, n. sp., in the enterocytes of a human patient with AIDS. J Protozoology 32: 250–254. 10.1111/j.1550-7408.1985.tb03046.x 4009510

[6] Keeling PJ (2003) Congruent evidence from alpha-tubulin and beta-tubulin gene phylogenies for a zygomycete origin of microsporidia. Fungal Genet Biol 38: 298–309. 12684019 10.1016/s1087-1845(02)00537-6

[7] Thomarat F, Vivares CP, Gouy M (2004) Phylogenetic analysis of the complete genome sequence of *Encephalitozoon cuniculi* supports the fungal origin of microsporidia and reveals a high frequency of fast-evolving genes. J Mol Evol 59: 780–791. 10.1007/s00239-004-2673-0 15599510

[8] Wittner M, Weiss LM (1999) The Microsporidia and Microsporidiosis. ASM Press.

[9] Weiss LM, Edlind TD, Vossbrinck CR, Hashimoto T (1998) Microsporidian molecular phylogeny: the fungal connection. J Eukarot Microbio 46: 17S–18S. 10519229

[10] Weiss LM, Becnel JJ (2014) Microsporidia: Pathogens of Opportunity: Wiley Blackwell.

[11] Capella-Gutiérrez S, Marcet-Houben M, Gabaldón T (2012) Phylogenomics supports microsporidia as the earliest diverging clade of sequenced fungi. BMC biology 10: 1. 22651672 10.1186/1741-7007-10-47 PMC3586952

[12] James TY, Pelin A, Bonen L, Ahrendt S, Sain D, et al (2013) Shared signatures of parasitism and phylogenomics unite Cryptomycota and Microsporidia. Current Biol 23: 1548–1553. 10.1016/j.cub.2013.06.057 23932404

[13] Corradi N (2015) Microsporidia: eukaryotic intracellular parasites shaped by gene loss and horizontal gene transfers. Ann Rev Microbiol 69: 167–183. 26195306 10.1146/annurev-micro-091014-104136

[14] Didier ES, Weiss LM (2011) Microsporidiosis: not just in AIDS patients. Curr Opin Infec Dis 24: 490–495. 21844802 10.1097/QCO.0b013e32834aa152 PMC3416021

[15] Stentiford G, Becnel J, Weiss L, Keeling P, Didier E, et al (2016) Microsporidia–Emergent Pathogens in the Global Food Chain. Trends Parasit 32:336–348. 10.1016/j.pt.2015.12.004 PMC4818719 26796229

[16] Dunn AM, Terry RS, Smith JE (2001) Transovarial transmission in the microsporidia. Adv Parasitol 48: 57–100. 11013755 10.1016/s0065-308x(01)48005-5

[17] Terry RS, Smith JE, Sharpe RG, Rigaud T, Littlewood DTJ, et al (2004) Widespread vertical transmission and associated host sex–ratio distortion within the eukaryotic phylum Microspora. Proc Royal Soc of Lond B: Biolog Sci 271: 1783–1789. 10.1098/rspb.2004.2793 PMC1691802 15315893

[18] Pasteur L (1870) Études sur la maladie des vers à soie [M]. Paris, Gauthier-Villars, successeur de Mallet-Bachelier 1870:148–168.

[19] Didier PJ, Didier ES, Orenstein JM, Shadduck JA (1991) Fine structure of a new human microsporidian, *Encephalitozoon hellem*, in culture. J Protozool 38: 502–507. 1920150 10.1111/j.1550-7408.1991.tb04824.x

[20] Orenstein JM (1991) Microsporidiosis in the acquired immunodeficiency syndrome. The J Parasitol 77: 843–864. 1779288

[21] Shadduck JA (1989) Human microsporidiosis and AIDS. Rev Infect Dis 11: 203–207. 2649958 10.1093/clinids/11.2.203

[22] Weber R, Bryan RT, Schwartz DA, Owen RL (1994) Human microsporidial infections. Clin Micro Rev 7: 426–461. 7834600 10.1128/cmr.7.4.426 PMC358336

[23] Weidner E (1976) The microsporidian spore invasion tube. The ultrastructure, isolation, and characterization of the protein comprising the tube. J Cell Biol 71: 23–34. 10309 10.1083/jcb.71.1.23 PMC2109716

[24] Thelohan P (1892) Observations sur les Myxosporidies et essai de classification de ces organismes. Bull Soc Philomat de Par 4:165–178.

[25] Thelohan P (1894) Sur la presence d’une capsule a filament dans les spores des microsporidies. CR Acad Sci 118: 1425–1427.

[26] Ohshima K (1937) On the function of the polar filament of *Nosema bombycis*. Parasitol 29: 220–224.

[27] Weidner E (1972) Ultrastructural study of microsporidian invasion into cells. Zeitschrift für Parasitenkunde 40: 227–242. 4346238 10.1007/BF00329623

[28] Frixione E, Ruiz L, Santillán M, de Vargas LV, Tejero JM, et al (1992) Dynamics of polar filament discharge and sporoplasm expulsion by microsporidian spores. Cell Motil Cytoskel 22: 38–50.

[29] Delbac F, Peuvel I, Metenier G, Peyretaillade E, Vivares CP (2001) Microsporidian invasion apparatus: identification of a novel polar tube protein and evidence for clustering of ptp1 and ptp2 genes in three Encephalitozoon species. Infect Immun 69: 1016–1024. 10.1128/IAI.69.2.1016-1024.2001 11159998 PMC97982

[30] Peuvel I, Peyret P, Metenier G, Vivares CP, Delbac F (2002) The microsporidian polar tube: evidence for a third polar tube protein (PTP3) in *Encephalitozoon cuniculi*. Mol Biochem Parasitol 122: 69–80. 12076771 10.1016/s0166-6851(02)00073-7

[31] Delbac F, Peyret P, Metenier G, David D, Danchin A, et al (1998) On proteins of the microsporidian invasive apparatus: complete sequence of a polar tube protein of *Encephalitozoon cuniculi*. Mol Microbiol 29: 825–834. 9723921 10.1046/j.1365-2958.1998.00975.x

[32] Weiss LM, Delbac F, Russell Hayman J, Pan G, Dang X, et al (2014) The Microsporidian Polar Tube and Spore Wall In: Microsporidia: Pathogens of Opportunity (Weiss LM, Becnell JJ Eds) Wiley Blackwell, pp 261–306.

[33] Keohane EM, Orr GA, Zhang HS, Takvorian PM, Cali A, et al (1998) The molecular characterization of the major polar tube protein gene from *Encephalitozoon hellem*, a microsporidian parasite of humans. Mol Biochem Parasitol 94: 227–236. 9747973 10.1016/s0166-6851(98)00071-1 PMC3109642

[34] Bouzahzah B, Weiss LM (2010) Glycosylation of the major polar tube protein of *Encephalitozoon cuniculi*. Parasitol Res 107: 761–764. 10.1007/s00436-010-1950-7 20556427 PMC6716521

[35] Xu Y, Takvorian PM, Cali A, Orr G, Weiss LM (2004) Glycosylation of the major polar tube protein of *Encephalitozoon hellem*, a microsporidian parasite that infects humans. Infect Immun 72: 6341–6350. 10.1128/IAI.72.11.6341-6350.2004 15501763 PMC523040

[36] Peek R, Delbac F, Speijer D, Polonais V, Greve S, et al (2005) Carbohydrate moieties of microsporidian polar tube proteins are targeted by immunoglobulin G in immunocompetent individuals. Infect Immun 73: 7906–7913. 10.1128/IAI.73.12.7906-7913.2005 16299281 PMC1307029

[37] Polonais V, Prensier G, Méténier G, Vivarès CP, Delbac F (2005) Microsporidian polar tube proteins: highly divergent but closely linked genes encode PTP1 and PTP2 in members of the evolutionarily distant Antonospora and Encephalitozoon groups. Fungal Genetics Biol 42: 791–803. 16051504 10.1016/j.fgb.2005.05.005

[38] Dolgikh VV, Semenov PB (2003) The spore wall and polar tube proteins of the microsporidian *Nosema grylli*: the major spore wall protein is released before spore extrusion. Tsitologiia 45: 324–329. 14520889

[39] Bouzahzah B, Nagajyothi F, Ghosh K, Takvorian PM, Cali A, et al (2010) Interactions of *Encephalitozoon cuniculi* polar tube proteins. Infect Immun 78: 2745–2753. 10.1128/IAI.01205-09 20308291 PMC2876541

[40] Xu Y, Takvorian P, Cali A, Weiss LM (2003) Lectin binding of the major polar tube protein (PTP1) and its role in invasion. J Eukaryot Microbiol 50 Suppl: 600–601. 14736177 10.1111/j.1550-7408.2003.tb00644.x

[41] Polonais V, Belkorchia A, Roussel M, Peyretaillade E, Peyret P, et al (2013) Identification of two new polar tube proteins related to polar tube protein 2 in the microsporidian *Antonospora locustae*. FEMS Microbiol Lett 346: 36–44. 10.1111/1574-6968.12198 23763358

[42] Slamovits CH, Fast NM, Law JS, Keeling PJ (2004) Genome compaction and stability in microsporidian intracellular parasites. Current Biology 14: 891–896. 10.1016/j.cub.2004.04.041 15186746

[43] Brosson D, Kuhn L, Delbac F, Garin J, P Vivarès C, et al (2006) Proteomic analysis of the eukaryotic parasite *Encephalitozoon cuniculi* (microsporidia): a reference map for proteins expressed in late sporogonial stages. Proteomics 6: 3625–3635. 10.1002/pmic.200500796 16691553

[44] Yang D, Pan G, Dang X, Shi Y, Li C, et al (2015) Interaction and assembly of two novel proteins in the spore wall of the microsporidian species *Nosema bombycis* and their roles in adherence to and infection of host cells. Infect Immun 83: 1715–1731. 10.1128/IAI.03155-14 25605761 PMC4363453

[45] Peuvel-Fanget I, Polonais V, Brosson D, Texier C, Kuhn L, et al (2006) EnP1 and EnP2, two proteins associated with the *Encephalitozoon cuniculi* endospore, the chitin-rich inner layer of the microsporidian spore wall. Int J Parasitol 36: 309–318. 10.1016/j.ijpara.2005.10.005 16368098

[46] Wu Z, Li Y, Pan G, Zhou Z, Xiang Z (2009) SWP25, a novel protein associated with the *Nosema bombycis* endospore. J Eukaryot Microbiol 56: 113–118. 10.1111/j.1550-7408.2008.00375.x 19457051

[47] Weidner E (1982) The microsporidian spore invasion tube. III. Tube extrusion and assembly. J Cell Biol 93: 976–979. 7119008 10.1083/jcb.93.3.976 PMC2112143

[48] De Boer P, Hoogenboom JP, Giepmans BN (2015) Correlated light and electron microscopy: ultrastructure lights up! Nat Methods 12: 503–513. 10.1038/nmeth.3400 26020503

[49] Moore CL, Cheng D, Shami GJ, Murphy CR (2016) Correlated light and electron microscopy observations of the uterine epithelial cell actin cytoskeleton using fluorescently labeled resin-embedded sections. Micron 84: 61–66. 10.1016/j.micron.2016.02.010 26930006

[50] Foucault C, Drancourt M (2000) Actin mediates *Encephalitozoon intestinalis* entry into the human enterocyte-like cell line, Caco-2. Microb Pathog 28: 51–58. 10.1006/mpat.1999.0329 10644491

[51] Bohne W, Bottcher K, Gross U (2011) The parasitophorous vacuole of *Encephalitozoon cuniculi*: biogenesis and characteristics of the host cell-pathogen interface. Int J Med Microbiol 301: 395–399. 10.1016/j.ijmm.2011.04.006 21550847

[52] Magaud A, Achbarou A, DesportesLivage I (1997) Cell invasion by the microsporidium *Encephalitozoon intestinalis*. J Eukary Microbiol 44. 10.1111/j.1550-7408.1997.tb05795.x 9508462

[53] Schottelius J, Schmetz C, Kock NP, Schuler T, Sobottka I, et al (2000) Presentation by scanning electron microscopy of the life cycle of microsporidia of the genus *Encephalitozoon*. Microbes Infect 2: 1401–1406. 11099925 10.1016/s1286-4579(00)01293-4

[54] Bigliardi E, Sacchi L (2001) Cell biology and invasion of the microsporidia. Microbes Infect 3: 373–379. 11369274 10.1016/s1286-4579(01)01393-4

[55] Weidner E, Byrd W (1982) The microsporidian spore invasion tube. II. Role of calcium in the activation of invasion tube discharge. J Cell Biol 93: 970–975. 6811603 10.1083/jcb.93.3.970 PMC2112170

[56] Qian ZM, Li H, Sun H, Ho K (2002) Targeted drug delivery via the transferrin receptor-mediated endocytosis pathway. Pharmacol Rev 54: 561–587. 12429868 10.1124/pr.54.4.561

[57] Chen C, Paw BH (2012) Cellular and mitochondrial iron homeostasis in vertebrates. Biochimica et Biophysica Acta (BBA)-Molecular Cell Research 1823: 1459–1467. 22285816 10.1016/j.bbamcr.2012.01.003 PMC3350831

[58] Martin DN, Uprichard SL (2013) Identification of transferrin receptor 1 as a hepatitis C virus entry factor. PNAS 110: 10777–10782. 10.1073/pnas.1301764110 23754414 PMC3696786

[59] Radoshitzky SR, Abraham J, Spiropoulou CF, Kuhn JH, Nguyen D, et al (2007) Transferrin receptor 1 is a cellular receptor for New World haemorrhagic fever arenaviruses. Nature 446: 92–96. 10.1038/nature05539 17287727 PMC3197705

[60] Hueffer K, Palermo LM, Parrish CR (2004) Parvovirus infection of cells by using variants of the feline transferrin receptor altering clathrin-mediated endocytosis, membrane domain localization, and capsid-binding domains. J Virol 78: 5601–5611. 10.1128/JVI.78.11.5601-5611.2004 15140957 PMC415789

[61] Parker JS, Murphy WJ, Wang D, O'Brien SJ, Parrish CR (2001) Canine and feline parvoviruses can use human or feline transferrin receptors to bind, enter, and infect cells. J Virol 75: 3896–3902. 10.1128/JVI.75.8.3896-3902.2001 11264378 PMC114880

[62] Ross SR, Schofield JJ, Farr CJ, Bucan M (2002) Mouse transferrin receptor 1 is the cell entry receptor for mouse mammary tumor virus. PNAS 99: 12386–12390. 10.1073/pnas.192360099 12218182 PMC129454

[63] Suetake T, Tsuda S, Kawabata S, Miura K, Iwanaga S, et al (2000) Chitin-binding proteins in invertebrates and plants comprise a common chitin-binding structural motif. J Biol Chem 275: 17929–17932. 10.1074/jbc.C000184200 10770921

[64] Cali A, Weiss LM, Takvorian PM (2002) *Brachiola algerae* spore membrane systems, their activity during extrusion, and a new structural entity, the multilayered interlaced network, associated with the polar tube and the sporoplasm. J Eukaryot Microbiol 49: 164–174. 12043963 10.1111/j.1550-7408.2002.tb00361.x

[65] Frixione E, Ruiz L, Cerbon J, Undeen AH (1997) Germination of *Nosema algerae* (Microspora) spores: conditional inhibition by D2O, ethanol and Hg2+ suggests dependence of water influx upon membrane hydration and specific transmembrane pathways. J Eukaryot Microbiol 44: 109–116. 9190262 10.1111/j.1550-7408.1997.tb05946.x

[66] Ronnebaumer K, Gross U, Bohne W (2008) The nascent parasitophorous vacuole membrane of *Encephalitozoon cuniculi* is formed by host cell lipids and contains pores which allow nutrient uptake. Eukaryot Cell 7: 1001–1008. 10.1128/EC.00004-08 18408058 PMC2446663

[67] Fasshauer V, Gross U, Bohne W (2005) The parasitophorous vacuole membrane of *Encephalitozoon cuniculi* lacks host cell membrane proteins immediately after invasion. Eukaryot Cell 4: 221–224. 10.1128/EC.4.1.221-224.2005 15643077 PMC544160

[68] Tomita T, Bzik DJ, Ma YF, Fox BA, Markillie LM, et al (2013) The *Toxoplasma gondii* cyst wall protein CST1 is critical for cyst wall integrity and promotes bradyzoite persistence. PLoS Pathog 9: e1003823 10.1371/journal.ppat.1003823 24385904 PMC3873430

[69] Graczyk TK, Johansson MA, Tamang L, Visvesvara GS, Moura LS, DaSilva AJ, Girouard AS, Matos O. (2007) Retrospective species identification of microsporidian spores in diarrheic fecal samples from human immunodeficiency virus/AIDS patients by multiplexed fluorescence in situ hybridization. J Clin Microbiol. 45(4):1255–60. 10.1128/JCM.01975-06 17287331 PMC1865804

[70] Hester JD, Lindquist HD, Bobst AM, Schaefer FW 3rd. (2000) Fluorescent in situ detection of *Encephalitozoon hellem* spores with a 6-carboxyfluorescein-labeled ribosomal RNA-targeted oligonucleotide probe. J Eukaryot Microbiol. 47(3):299–308. 10847348 10.1111/j.1550-7408.2000.tb00051.x

