# Supplementary material for: The role of microsporidian polar tube protein 4 (PTP4) in host cell infection
Source: PLoS Pathog. 2017 Apr 20;13(4):e1006341. doi: 10.1371/journal.ppat.1006341 (PMC5413088; doi:10.1371/journal.ppat.1006341)
Supplement: S1 Text — (DOCX) [file ppat.1006341.s001.docx]

**Supporting Information**

**Immunoprecipitation of native EhPTP4 from spore lysis by MAb-EhPTP4 and PcAb-EhPTP4.**

Immunoprecipitation (IP) was utilized to further prove the interaction of native EhPTP4 with the EhPTP4 monoclonal (Mab-PTP4) and polyclonal (rab-PcPTP4) antibodies. Briefly, RK13 cells were cultured in a T75 flask (Corning) to confluence and then infected with 1×10^7^ spores. The cells were harvested two weeks post-infection and cell lysates were obtained by a modified procedure as reported previously [34]. Infected RK13 cells were spun down and disrupted in RIPA buffer (Sigma-Aldrich) containing protease inhibitor (Protease inhibitor cocktail; Thermo Fisher Scientific) with 0.4 g 0.5 μm acid-washed glass beads (Sigma-Aldrich) for 1 min on a Mini-Beadbeater (BioSpec Products). The disrupted host cell suspension was then clarified by centrifugation at 12000 rpm for 10 min.

For immunoprecipitation, 50 μl of Protein G agarose beads in PBS was incubated with 20 μl of MAb-EhPTP4 or mouse negative IgG at 4°C for 1hr. The protein G agarose beads conjugates were washed three times with PBS and resuspended in a final volume of 500 μl cell lyses collected above. The samples were incubated at 4°C for 2hrs with gentle shaking. The samples were then centrifuged, and the pellets were washed three times with PBS. Fifty μl of 2X protein sample buffer (0.5M Tris-HCl pH 6.8, 4.4% SDS, 20% glycerol, 2% 2-mercaptoethanol and 0.01% bromophenol blue) was added to each sample and the samples were then boiled for 5 min. Following centrifugation the supernatant was loaded onto an SDS-PAGE gel, transferred to a PVDF membrane. Immunoblot analysis was then performed using rab-PcAb-EhPTP4 at a dilution of 1:500. A reciprocal Co-IP assay was also performed with rab-PcAb-EhPTP4 and the blot was probed with MAb-EhPTP4 at a dilution of 1:100.
