# Supplementary figures and images for: The role of microsporidian polar tube protein 4 (PTP4) in host cell infection
Source: PLoS Pathog. 2017 Apr 20;13(4):e1006341. doi: 10.1371/journal.ppat.1006341 (PMC5413088; doi:10.1371/journal.ppat.1006341)

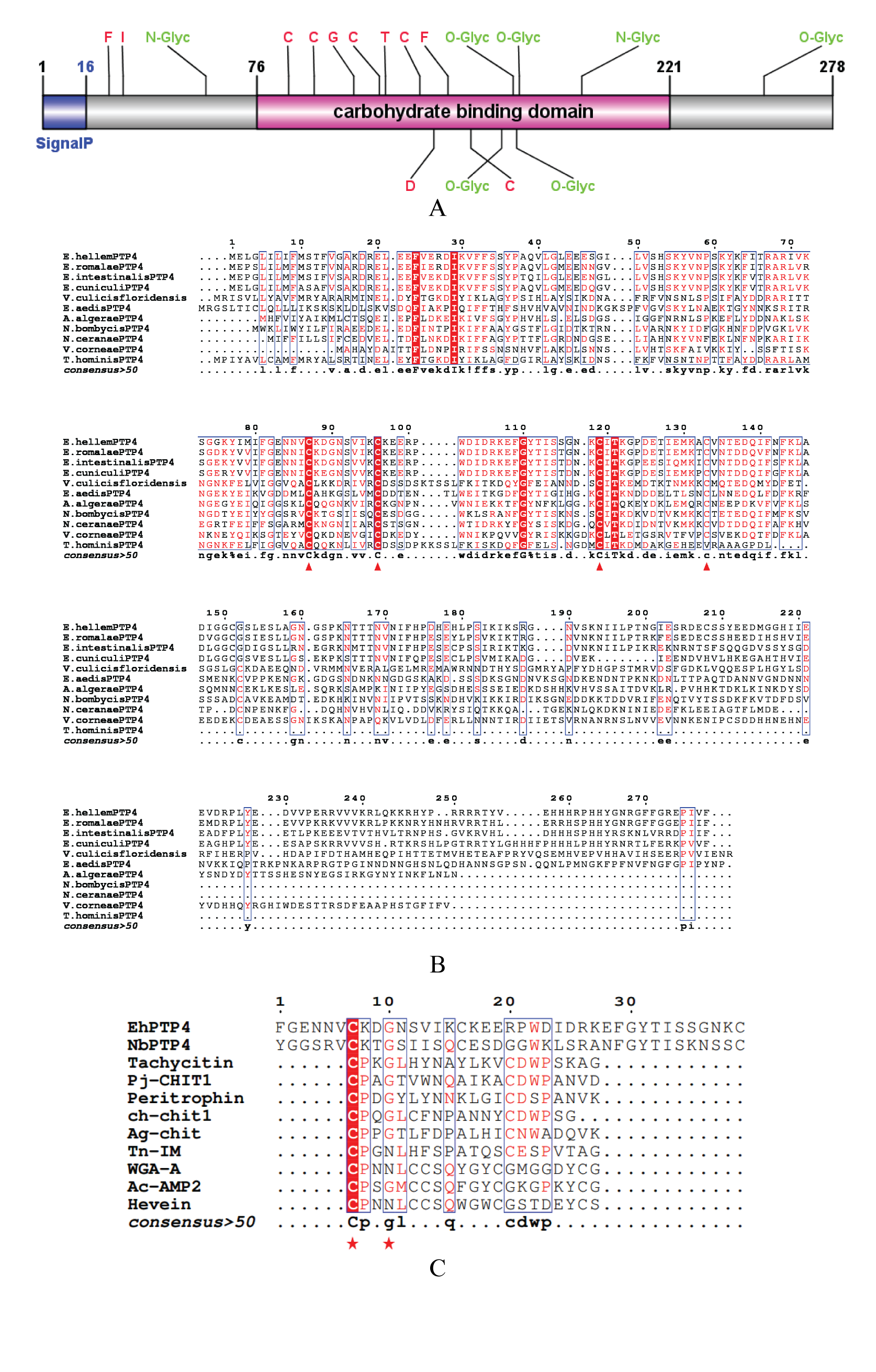

Supplement: S1 Fig — (A) Diagram of protein sequence of EhPTP4. A carbohydrate binding domain (chitin binding domain and cellulose binding domain) predicted by SMART (http://smart.embl-heidelberg.de/) are indicated. Highly conserved residues among PTP4 homologs are shown in red. N-glycosylation sites and O-glycosylation sites are shown in green. A signal peptide predicted in EhPTP4 is shown in blue. (B) Multiple-sequence alignment of EhPTP4 and homologs. Four cysteine residues were highly conserved among PTP4 homologous (red triangles). The N-terminal of PTP4 homologs was relatively highly conserved compared to the C-terminal. (C) Chitin binding domain analysis of PTP4. Cys, Pro, and Gly have significant influence on the structure constructions of chitin-binding proteins. Cys87 and Gly90 of EhPTP4 are conserved among known chitin binding proteins (red stars), revealed that EhPTP4 may be also a chitin binding protein. Tachycitin: T. tridentatus tachycitin. Pj-CHIT1: Penaeus japonica chitinase 1. Peritrophin: 44-kDa glycoprotein from Lucilia cuprina. Ch-chit1: Chelonus sp. Chitinase. Ag-chit: Anopheles gambiae chitinase. Tn-IM: Trichoplusia ni intestinal mucin. WGA-A: Wheat germ agglutinin. Ac-AMP2: Amaranthus caudatus antimicrobial protein 2. Hevein: Hevein from rubber tree. (TIF) [file ppat.1006341.s002.tif]

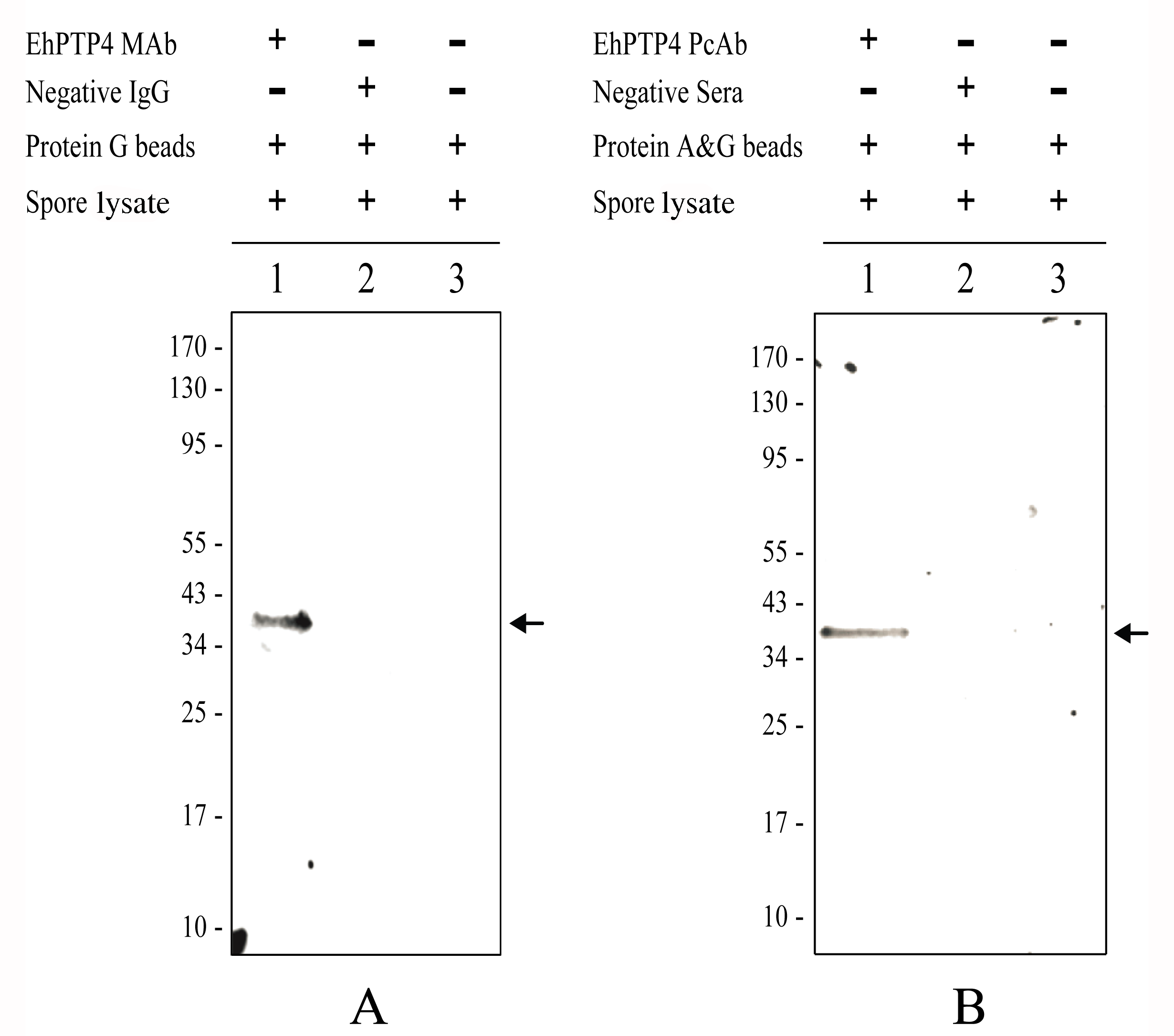

Supplement: S2 Fig — (A) Mouse serum (preimmunization) was used as negative control. Arrowhead indicates the native EhPTP4 band pulled down by MAb-EhPTP4 and probed with rab-PcAb EhPTP4. (B) Rabbit serum (preimmunization) was used as negative control. Arrowhead indicates the native EhPTP4 band pulled down by rab-PcAb EhPTP4 and probed with MAb-Ab EhPTP4. (TIF) [file ppat.1006341.s003.tif]

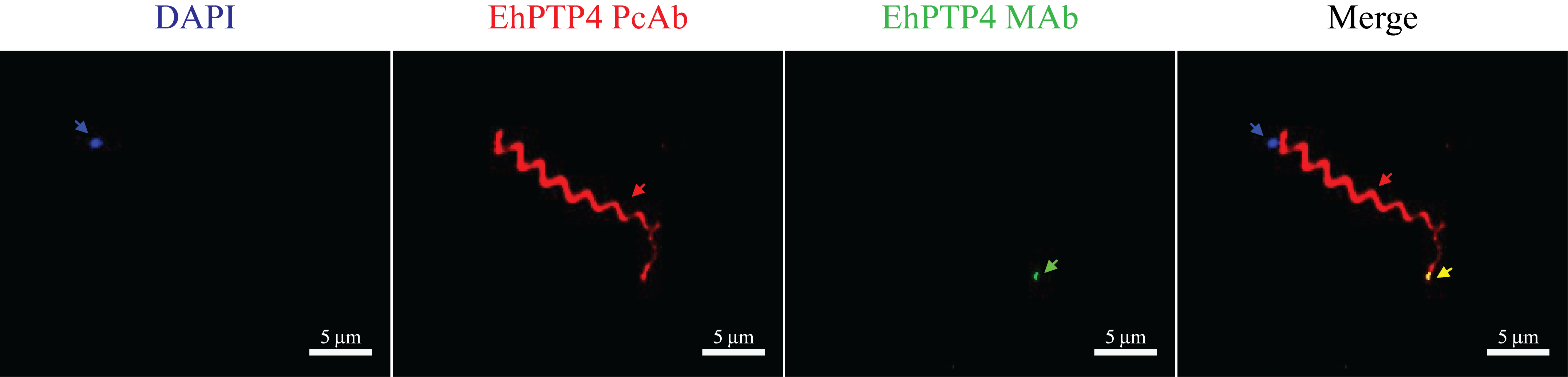

Supplement: S3 Fig — Extruded polar tubes of E. hellem were initially incubated with rab-PcAb-EhPTP4 and MAb-EhPTP4 detected by anti-rabbit Alexa Fluor 594 secondary antibody (red) and anti-mouse Alexa Fluor 488 secondary antibody (green). Blue arrows indicate the labeling of the nuclei of E. hellem; Red arrows indicate the labeling of the entire polar tube by rab-PcAb-EhPTP4; Green arrows indicate labeling of the tip of polar tube by MAb-EhPTP4; Yellow arrows indicate the merged signal of the rab-Pc EhPTP4 and MAb-EhPTP4 staining. Bar, 5 μm. (TIF) [file ppat.1006341.s004.tif]

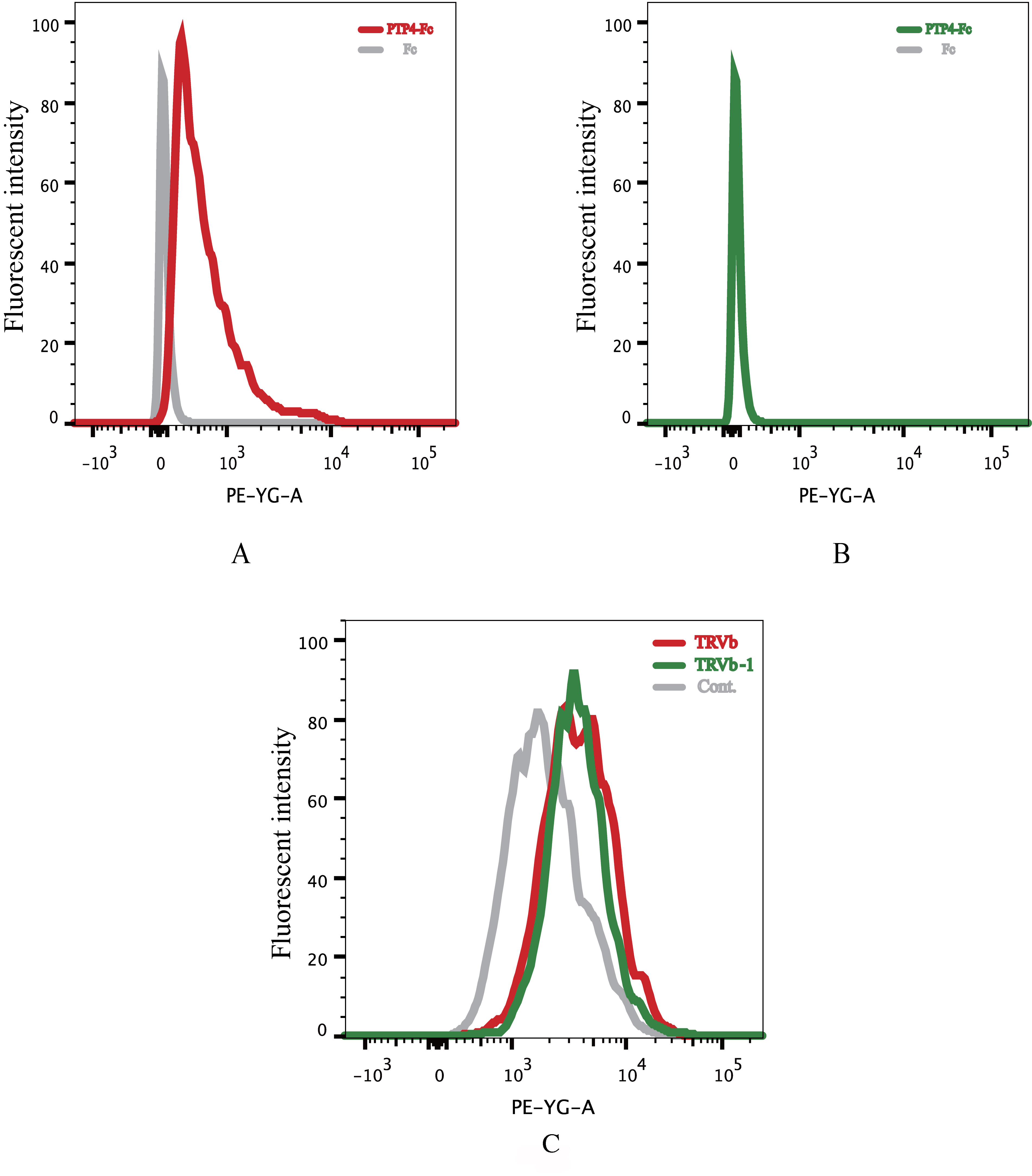

Supplement: S4 Fig — HFF cells were detached by treating with 1X Citric saline solution and incubated with EhPTP4-Fc fusion proteins or Fc proteins. The curve of EhPTP4 (Red) is significantly shifted compared to Fc curve (grey), revealed that EhPTP4 could bind to HFF cells (A). However, neither EhPTP4-Fc nor Fc protein could bind to the trypsinized cells on which the surface proteins were removed by trypsinization (B). The transferrin receptor 1 (TfR1) knockout CHO cell line TRVb and a CHO cell line TRVb-1 which expresses human transferrin receptor 1 (huTfR1) were used to examine binding of EhPTP4 to these cells. EhPTP4 was able to bind to both TRVb or TRVb-1 cells (C). This suggests that, in addition to TfR1, EhPTP4 can interact with other host cell proteins or post translational modifications on the host cell surface. (TIF) [file ppat.1006341.s005.tif]

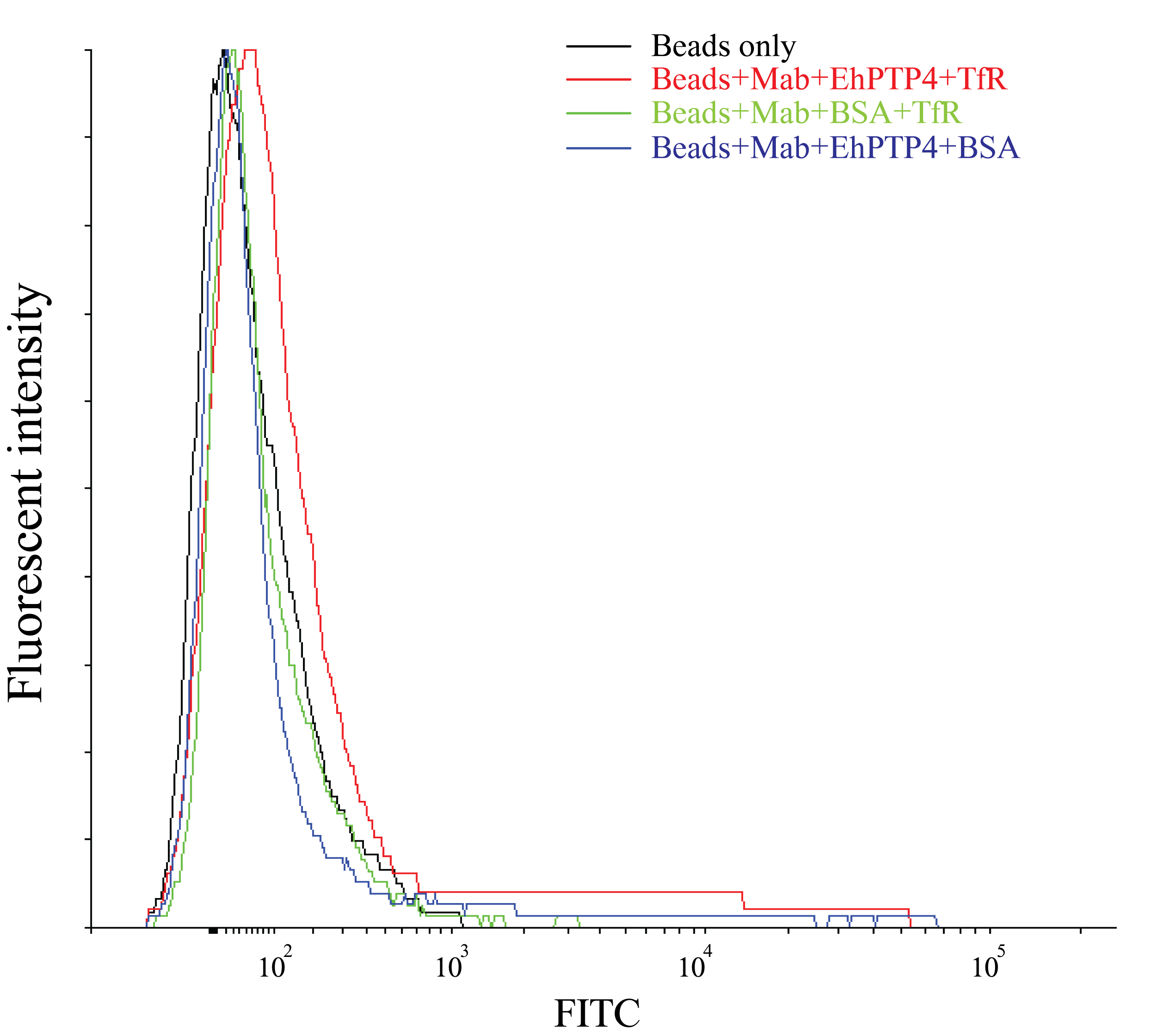

Supplement: S5 Fig — An immuno-pull-down assay was performed using MAb-EhPTP4 conjugated protein G sepharose beads which were then incubated with FITC conjugated anti-human TfR1 mouse monoclonal antibody. The red curve demonstrates that the pull down using recTfR-1 and recEhPTP4 mixtures was significantly shifted compared to the pull down result from recTfR-1 and BSA (green curve) or recEhPTP4 and BSA mixtures (blue curve). This result was consistent with the pull down results demonstrated by immunoblot in Fig 4F. (TIF) [file ppat.1006341.s006.tif]

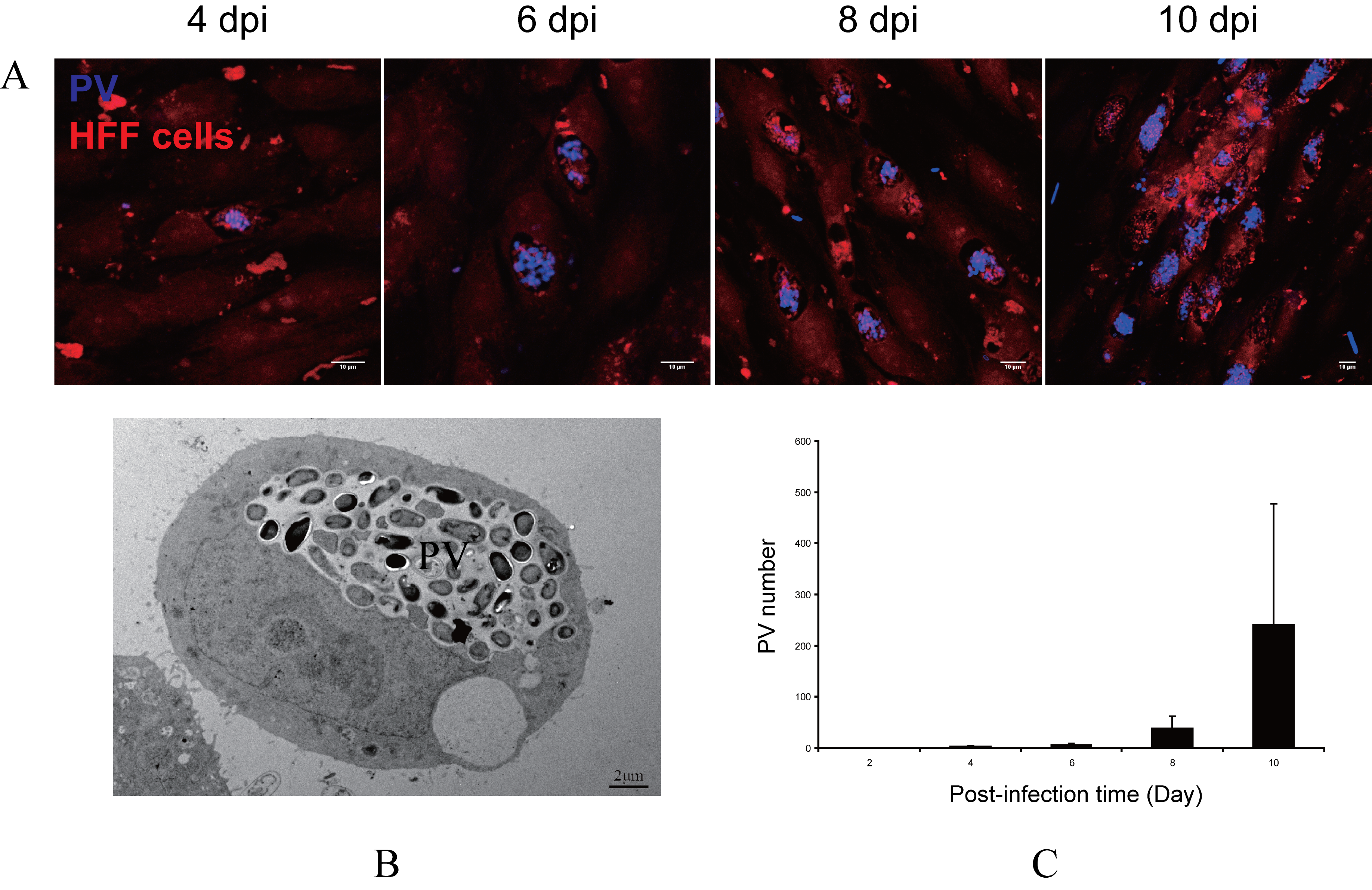

Supplement: S6 Fig — (A) Time dependent infection of E. hellem in HFF cells, mature spores could be identified starting at 3 days post-infection. The spore wall was stained with Calcofluor White (blue), cells were stained with GelRed (red). (B) TEM of a microsporidian parasitophorous vacuole (PV) in HFF cells at 6 days post-infection. (C) Time dependent in vitro growth curve of visible E. hellem PVs in HFF cells. (TIF) [file ppat.1006341.s007.tif]
