# Supplementary material for: The role of microsporidian polar tube protein 4 (PTP4) in host cell infection
Source: PLoS Pathog. 2017 Apr 20;13(4):e1006341. doi: 10.1371/journal.ppat.1006341 (PMC5413088; doi:10.1371/journal.ppat.1006341)
Supplement: S1 Table — (DOC) [file ppat.1006341.s008.doc]

**Table S1. List of primers used in this study**

| Primers | Sequences of oligonucleotides（5’→3’） |
| --- | --- |
| pMCSG7-Forward | TAACATGGAAGTGGATAACG |
| pMCSG7-Reverse | TGGATTGGAAGTACAGGTTC |
| pMCSG7-EhPTP4-Forward | GAACCTGTACTTCCAATCCAATGGAACTGGGTTTGATCTT |
| pMCSG7-EhPTP4-Reverse | CGTTATCCACTTCCATGTTACTAGAAAACTATGGGCTCTC |
| pFUSE-Fc-Forward | GACAAAACTCACACATGCCC |
| pFUSE-Fc-Reverse | AGATCTAACCATGGCCGATA |
| pFUSE-EhPTP4-Forward | TATCGGCCATGGTTAGATCTAAGGACAGGGAGCTGGAAGA |
| pFUSE-EhPTP4-Reverse | GGCATGTGTGAGTTTTGTCGAAAACTATGGGCTCTCTTCC |
